# An Optimized Flow Cytometric Method to Demonstrate the Differentiation Stage-Dependent Ca^2+^ Flux Responses of Peripheral Human B Cells

**DOI:** 10.3390/ijms24109107

**Published:** 2023-05-22

**Authors:** Anna Bajnok, Timea Serény-Litvai, Viktória Temesfői, Jasper Nörenberg, Róbert Herczeg, Ambrus Kaposi, Timea Berki, Emese Mezosi

**Affiliations:** 1 Department of Obstetrics and Gynecology, Clinical Center, Medical School, University of Pécs, 7624 Pécs, Hungary; bajnok.anna@pte.hu (A.B.); jasper.norenberg@pte.hu (J.N.); 2 National Laboratory on Human Reproduction, University of Pécs, 7624 Pécs, Hungary; 3 Szentágothai Research Centre, University of Pécs, 7624 Pécs, Hungary; 4 Department of Immunology and Biotechnology, Clinical Center, Medical School, University of Pécs, 7624 Pécs, Hungary; 5 Department of Laboratory Medicine, Clinical Center, Medical School, University of Pécs, 7624 Pécs, Hungary; 6 Department of Medical Microbiology and Immunology, Clinical Center, Medical School, University of Pécs, 7624 Pécs, Hungary; 7 Bioinformatics Research Group, Genomics and Bioinformatics Core Facility, University of Pécs, 7624 Pécs, Hungary; 8 Department of Programming Languages and Compilers, Faculty of Informatics, Eötvös Loránd University, 1053 Budapest, Hungary; 9 First Department of Internal Medicine, Clinical Center, Medical School, University of Pécs, 7624 Pécs, Hungary

**Keywords:** B-cell activation, calcium flux response, BCR responsivity, B-lymphocyte subsets

## Abstract

Calcium (Ca^2+^) flux acts as a central signaling pathway in B cells, and its alterations are associated with autoimmune dysregulation and B-cell malignancies. We standardized a flow-cytometry-based method using various stimuli to investigate the Ca^2+^ flux characteristics of circulating human B lymphocytes from healthy individuals. We found that different activating agents trigger distinct Ca^2+^ flux responses and that B-cell subsets show specific developmental-stage dependent Ca^2+^ flux response patterns. Naive B cells responded with a more substantial Ca^2+^ flux to B cell receptor (BCR) stimulation than memory B cells. Non-switched memory cells responded to anti-IgD stimulation with a naive-like Ca^2+^ flux pattern, whereas their anti-IgM response was memory-like. Peripheral antibody-secreting cells retained their IgG responsivity but showed reduced Ca^2+^ responses upon activation, indicating their loss of dependence on Ca^2+^ signaling. Ca^2+^ flux is a relevant functional test for B cells, and its alterations could provide insight into pathological B-cell activation development.

## 1. Introduction

Alteration in the Ca^2+^ flux response of B cells is associated with B-cell dysfunction, skewed humoral immune responses, autoimmune disorders, and B-cell malignancies [1,2,3]. Investigating the Ca^2+^ flux characteristics of peripheral human B cells to various stimuli may provide valuable insight into the activation process of B lymphocytes in health and disease. The intracellular Ca^2+^ concentration of lymphocytes results from carefully balanced active transport and gradient-driven changes influenced by multiple receptors and environment-sensing pathways [4]. The dynamic changes in the Ca^2+^ concentration act as a central signaling pathway capable of encoding and transducing a wide range of information, from apoptosis through tolerance to activation [2,5]. The distinct subsets of B cells have specific differences in how they react to signals and how the generated Ca^2+^ flux is decoded. The diversity in responsiveness is directed by the surface receptor repertoire and the structure and intracellular environment of the BCR. The maturity of the cells fundamentally determines their ability to react, respond, differentiate, and survive [6,7].

In B lymphocytes, different essential surface receptors may initiate Ca^2+^ mobilization. The BCR is a membrane-bound Ig associated with other transmembrane proteins, and the genes encoding the immunoglobulin heavy-chain and light-chain proteins undergo constant recombination and maturation during B-cell development [8]. Upon BCR ligation, the associated CD79a and b are phosphorylated by the Src-family kinase (SFK) Lyn [9,10,11], leading to spleen tyrosine kinase (Syk) binding and phosphorylation [12]. Bruton’s tyrosine kinase (Btk) is activated, which phosphorylates phospholipase C-gamma 2 (PLCγ2), leading to the cleavage of phosphoinositide PI(4,5)P2 and the release of inositol-1,4,5,-triphosphate (IP3) and diacylglycerol (DAG) [13,14]. IP3 binds to its receptor on the endoplasmic reticulum (ER), leading to an initial release of Ca^2+^ from the intracellular stores that triggers the calcium-release-activated (CRAC) channels. The opening of the CRAC channels generates a prolonged Ca^2+^ influx from the extracellular space, constituting the plateau phase of the activation curve [15,16]. B lymphocytes can also be activated in a polyclonal, BCR-independent manner through highly mitogenic pathogen-associated molecular patterns (PAMPs) or other innate non-mitogenic stimuli [17]. 

Immature B cells in the bone marrow express an IgM-type BCR on their cell surface [18]. Transitional B cells that leave the bone marrow begin co-expressing an IgD-type BCR along the IgM with identical specificity [19]. IgD expression gradually increases throughout the transitional stages and reaches high levels in mature naive cells [20]. The dual expression of IgD and IgM BCR on B cells has been a long-standing riddle, and the function of IgD is still not completely clear [21]. Following antigen encounter, Ig class-switching and differentiation into memory cells or plasma cells (PCs) are induced [22]. CD27^+^ B cells can be further classified based on their IgD expression into IgD^+^ non-switched (NSw) and IgD^−^ Ig class-switched (Sw) memory cells. Sw memory cells originate from the germinal center response and express IgG, IgA, or IgE. Despite the similarities to IgG-expressing Sw memory cells, there is also substantial evidence to indicate that NSw memory cells could be the human equivalent of murine circulating marginal zone B cells [23]. Another distinction between CD27^+^ IgD^−^ Sw and CD27^−^ IgD^−^ double-negative (DN) cells can be made. Sw and DN memory subsets can differentiate into IgD^−^ CD27^high^ CD38^high^ plasmablasts (PBs) and PCs. The distinction between PBs and PCs is not obvious, not even when using CD138, which is considered to be a mature PC marker. Therefore, following the recommendation of Sanz et al., this population will be referred to as circulating antibody-secreting cells (ASCs) [24].

**Figure 1:**
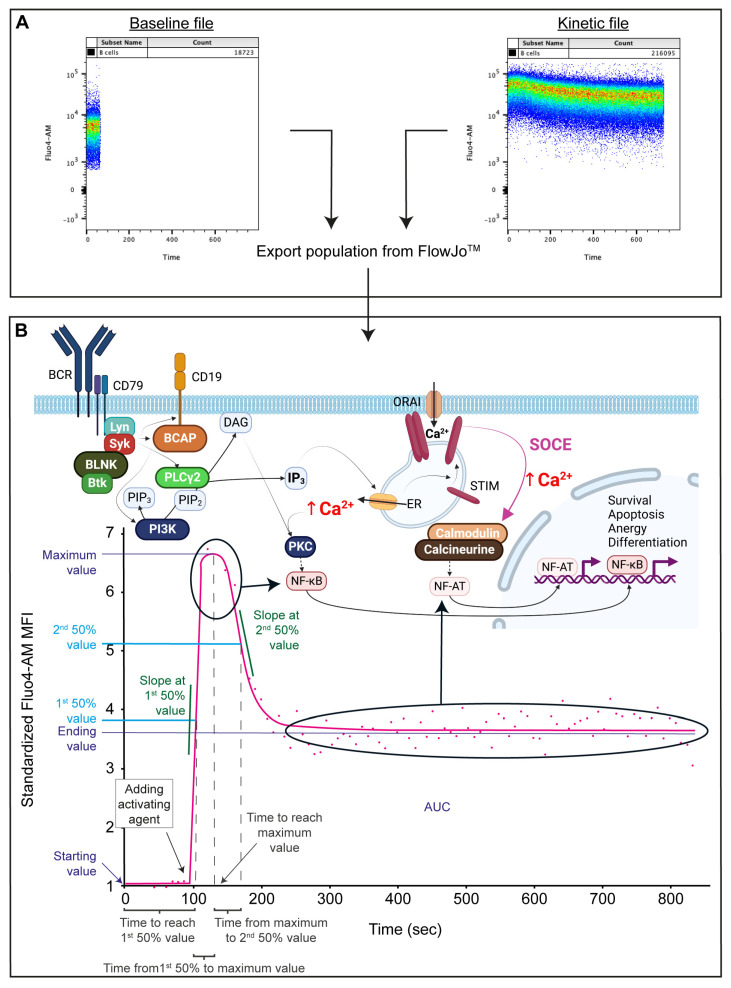
Calcium flux kinetics. (**A**) Export of gated B cell populations from baseline- and kinetic-file in FlowJo. (**B**) Simplified Ca^2+^ signaling pathway in B cells (upper panel) and schematic data output, visualizing the descriptive parameters generated by FacsKin. The tinted arrows indicate the relationship between specific parts of the activation curve and transcription factors. BCR: B-cell receptor, Lyn: Lck tyrosine kinase, Syk: spleen tyrosine kinase, BLNK: B-cell linker, Btk: Bruton’s tyrosine kinase, BCAP: B-cell adaptor for phosphoinositide 3-kinase, PLCγ2: phospholipase Cγ2, PIP_2_: phosphatidylinositol-4,5-diphosphate, PIP_3_: phosphatidylinositol-4,5-triphosphate, PI3K: phosphoinositide 3-kinase, DAG: diacylglycerol, IP_3_: inositol-1,4,5,-triphosphate, PKC: protein kinase C, NFκB: nuclear factor κB, ER: endoplasmic reticulum, STIM: stromal interaction molecule, NFAT: nuclear factor of activated T cells, SOCE: store-operated Ca^2+^ entry, AUC: area under the curve. Standardized Fluo-4 MFI: standardized Fluo-4-AM median fluorescence intensity, where the baseline absolute MFI value of Fluo-4 AM is standardized to 1 and relative parameter alterations are shown on the Y axis.

The Ca^2+^ signal can be considered the “master regulator” of most aspects of fate and function in B cells [1,3]. Emerging evidence indicates that modest alterations in BCR signaling are sufficient to promote autoimmunity in a B cell-intrinsic manner [3,25,26,27,28,29]. These observations have mainly been made in animal models of systemic autoimmunity. Several options are available for tracking the changes in the intracellular Ca^2+^ concentration of cells. Patch clamp methods allow for the precise characterization of Ca^2+^ flux on a single cell level [30] but do not provide the option to describe entire populations simultaneously. With the development of flow cytometric Ca^2+^-responsive probes, real-time monitoring of the Ca^2+^ flux response of selected cell populations became possible. For instance, Fluo-3 and Fluo-4 are both widely used fluorescent Ca^2+^ indicators that are excited by the 488 nm laser with an emission spectrum in the low 500 nm range, but Fluo-4 is brighter, permeates the cell membrane better, and has a large dynamic range for reporting Ca^2+^ [31]. Therefore, we aimed to optimize a Fluo4-AM-based flow cytometric method to monitor the real-time changes in intracellular Ca^2+^ flux in the first 12 min of the activation of peripheral human B cells (Figure 1). Although flow cytometric Ca^2+^ flux measurements are commonly used to describe the functional characteristics of cells [32,33,34], we combined Ca^2+^ flux measurements with a B-cell-specific surface staining panel that can be expanded and adapted to specific immunopathological questions. Using an algorithm developed previously by our collaborators and adapted to support this study [Facskin [35,36]], functions were fitted to the activation dynamics. We investigated how commonly used B cell-activating agents influence the Ca^2+^ flux pattern of circulating B lymphocyte subsets in healthy individuals. We tested the effect of anti-human IgG, IgM, IgG+M, ionomycin, CpG, phorbol 12-myristate 13-acetate (PMA), IL-4, anti-CD40, and lipopolysaccharide (LPS) on the Ca^2+^ mobilization of B-cell subsets. Our goal was to optimize a method that can be utilized to study human peripheral B-cell subsets in pathological conditions such as autoimmune disorders or B-cell malignancies and the effects of treatments.

## 2. Results

### 2.1. The Pattern of the Ca^2+^ Flux Kinetic Curves Corresponded with the Differentiation Stage of B Cell Subsets 

**Figure 2:**
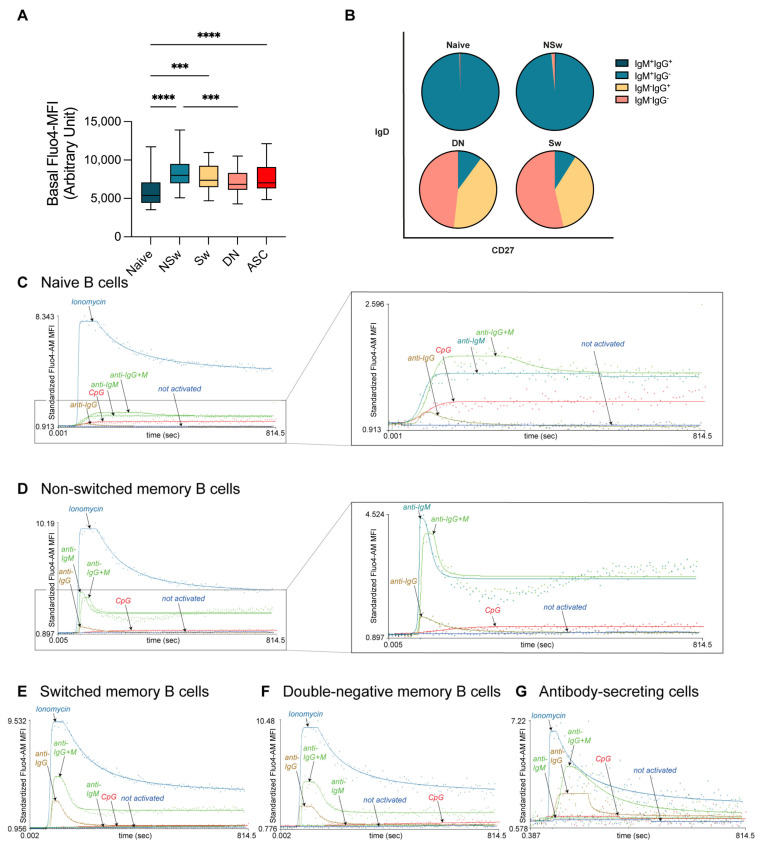
Immunoglobulin expression profile, baseline calcium level, and responsivity of B-cell subsets to various activating agents. (**A**) Baseline median fluorescence intensity (arbitrary unit) of Fluo-4 in B-cell subsets (*n* = 15) depicted as boxplots. The middle line shows the median and the whiskers represent the 5th to 95th percentile. Statistical comparison between the groups was performed by Friedman test and multiple comparisons FDR-corrected after Benjamini and Hochberg. (**B**) Prevalence (%) of surface-immunoglobulin expression in B-cell subsets. X-axis: CD27 expression, Y-axis: IgD expression. Representative calcium flux kinetic curves of the responsivity of naive (**C**), non-switched memory (**D**), switched memory (**E**), double-negative memory (**F**), antibody-secreting cells (**G**) to stimulation via IgG with anti-human F(ab’)_2_ specific anti-IgG, via IgM with F(ab’)_2_ anti-human Fc5µ specific anti-IgM, via IgG+M with anti-human F(ab’)_2_ IgG + IgM (H+L), CpG-B DNA, and ionomycin. X-axis: time (s), Y-axis: standardized Fluo-4-AM median fluorescence intensity, where the baseline absolute MFI value of Fluo-4 AM is standardized to 1 and relative parameter alterations are shown. *** *p* < 0.001, **** *p* < 0.0001, NSw: non-switched; Sw: switched, DN: double negative memory B cells, ASC: antibody-secreting cells.

**Figure 3:**
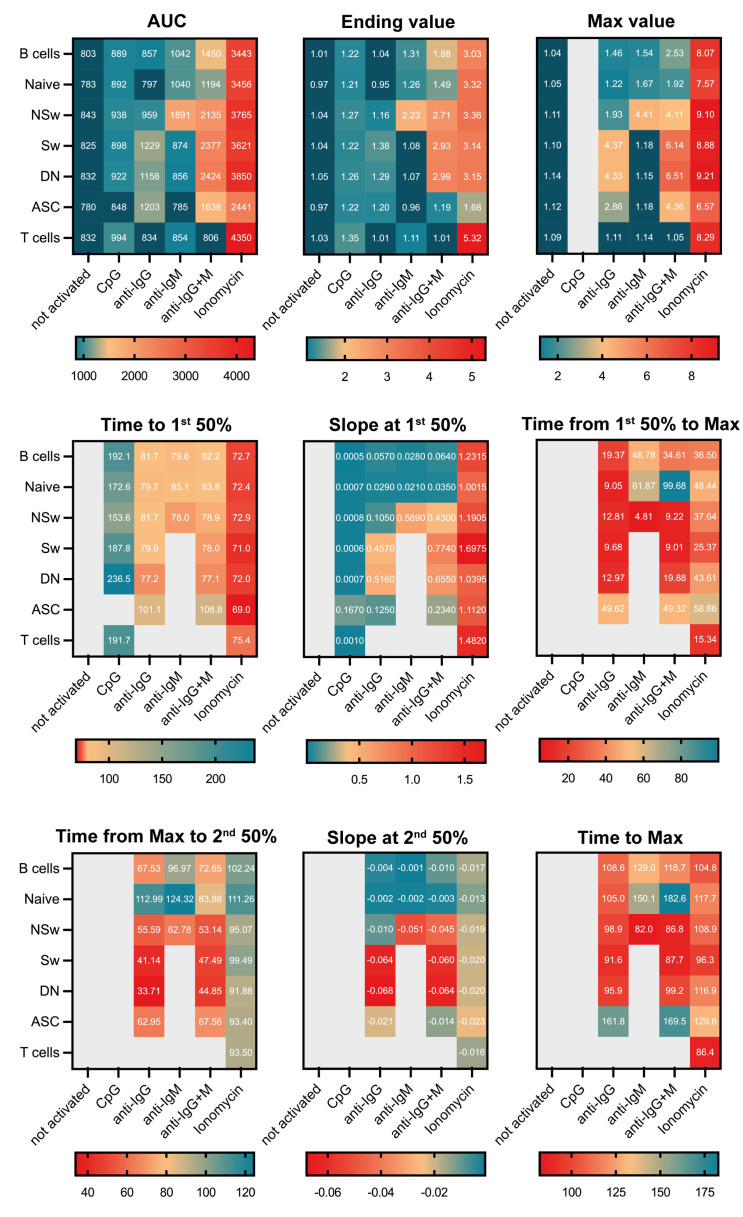
Heat map of all kinetics parameters. Heat map from the median values of kinetic curve parameters in B cells, T cells, and B-cell subsets in non-activated samples and response to CpG, anti-IgG, anti-IgM, anti-IgG+M, and ionomycin stimulation collected from 15 healthy individuals. The AUC: area under the curve, Ending, and Max parameters were expressed in arbitrary units, time parameters in seconds, and the slope parameters in MFI (median fluorescent intensity) change/second. Those parameters that cannot be calculated or are biologically irrelevant (i.e., T cells do not respond to anti-IgG+M) were left grey.

Naive B cells showed lower levels of Ca^2+^ prior to activation compared to post-antigen-experienced subsets (NSw, Sw, ASC, Figure 2A). Over 98% of naive B cells expressed only IgM; consequently, anti-IgM, anti-IgG+M, and ionomycin evoked a Ca^2+^ flux response. Ionomycin induced a higher and prompter Ca^2+^ response than IgM (higher AUC, Max, and Ending values and lower time to reach the 50% value, Figure 2B,C and Figure 3, Supplementary Table S1).

Within the memory subsets, NSw memory cells had the highest baseline Ca^2+^ level (Figure 2A). Over 97% of NSw memory cells expressed only IgM, and 1% expressed IgG along with IgM (Figure 2B). The NSw subset reacted to anti-IgM with a fast and intense response, which resembled the BCR-induced Ca^2+^ flux of IgG-expressing memory subsets (Sw and DN). Anti-IgG also slightly elevated the AUC and Max parameters in NSw cells compared to NA samples. However, as only around 1% of cells were shown to express IgG, this could be in part due to the cross-reactivity of the anti-IgG antibody to the IgM-type BCR. Ionomycin activation resulted in a higher and prompter Ca^2+^ response than IgM (higher AUC, Max, Ending values, 1st Slope, and lower time to reach 50%, Figure 2D, Supplementary Table S1).

Among Sw and DN memory cells, around 1/3 of cells expressed IgG, and around 9% expressed IgM, while over 50% had an IgM^−^ IgG^−^ phenotype, likely representing IgA-expressing memory cells (Figure 2B). Sw and DN memory cells responded to anti-IgG, anti-IgG+M, and ionomycin activation with similar Ca^2+^ flux curves. Ionomycin evoked a more robust and quicker Ca^2+^ flux compared to anti-IgG. Interestingly, the Ca^2+^ response produced by ionomycin resulted in a higher plateau phase, while the Ca^2+^ level after IgG binding declined quicker. Although about 10% of Sw and DN memory cells expressed only IgM, these subsets showed no response to anti-IgM stimulation (Figure 2E,F and Figure 3, Supplementary Table S1).

Little is known about the Ca^2+^ signaling of circulating antibody-secreting cells. In our experiments, ASCs reacted to anti-IgG, anti-IgG+M, and ionomycin activation but did not respond to anti-IgM. Anti-IgG and anti-IgG+M evoked elevated AUC and Max values compared to NA. Ionomycin activation resulted in higher AUC, Max, 1st Slope, and Ending and lower time to reach the 1st 50% values compared to NA and anti-IgG (Figure 2G and Figure 3, Supplementary Table S1).

**Figure 4:**
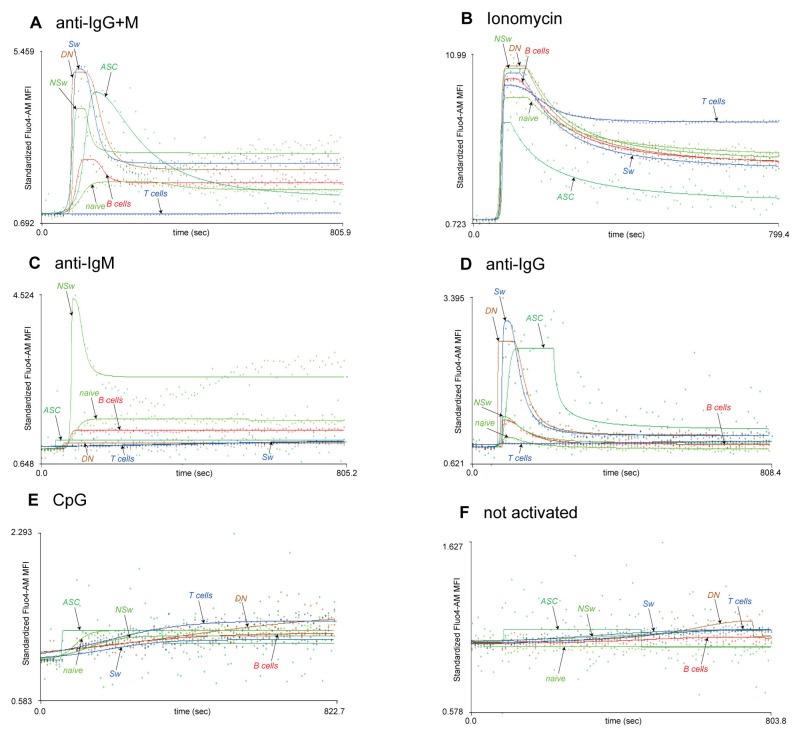
Comparison of the responsivity of B-cell subsets to each activating agent. Representative calcium flux kinetic curves of the responsivity of naive, NSw: non-switched memory, Sw: switched memory, DN: double-negative memory, and ASC: antibody-secreting cells to stimulation with anti-human F(ab’)_2_ IgG + IgM (H+L) (**A**), ionomycin (**B**), F(ab’)_2_ anti-human Fc5µ-specific anti-IgM (**C**), anti-human F(ab’)_2_ specific anti-IgG (**D**), and CpG-B DNA (**E**). The kinetic curves of samples where no activating agent was added are also presented for comparison (**F**). X-axis: time (s), Y-axis: standardized Fluo-4-AM median fluorescence intensity, where the baseline absolute MFI value of Fluo-4 AM is standardized to 1 and relative parameter alterations are shown.

Upon anti-IgG+M stimulation, all memory B-cell subsets reached higher AUC, Max, and Ending values than naive B cells. The Max Ca^2+^ level of NSw memory B cells remained lower compared to Sw and DN memory B cells (Figure 3 and Figure 4A, Supplementary Table S2).

ASCs are known to down-regulate their cell surface Ig expression [24]. Therefore, it is intriguing that we were able to stimulate peripheral ASCs via their IgG receptor. However, they did show a distinct Ca^2+^ flux pattern compared to memory subsets. Despite their IgG expression, their Max value remained lower than Sw and DN memory subsets, and they had a prolonged response to BCR stimulation. After the peak, the intracellular Ca^2+^ level decreased gradually and almost returned to the baseline by the end of the measurement without a clear plateau phase (Figure 4A). As the plateau phase of the Ca^2+^ flux is indispensable for *c-Myc* expression [2], which is repressed when B cells differentiate into ASCs [37], our findings align with physiological changes during B-cell differentiation. 

The ionomycin-induced Ca^2+^ mobilizing capacity of circulating antibody-secreting cells was lower compared to the other subsets (lower AUC, Max, and Ending values). No differences were found in the activation speed between the subsets after ionomycin stimulation. The max value of naive B cells was lower, and their 1st Slope was less steep than DN and Sw memory B cells (Figure 3 and Figure 4B, Supplementary Table S2).

When comparing the IgM responsivity of subsets, we found that non-switched memory cells responded stronger to anti-IgM activation than naive B cells. NSw memory cells had significantly higher AUC, Max, 1st Slope, and Ending and lower time to reach the 1st 50% values in response to anti-IgM stimulation than naive cells (Figure 4C, Supplementary Table S2).

CpG led to a moderate, gradual increase in the intracellular Ca^2+^ level of B-cell subsets. We found a gradual increase in the MFI of Fluo-4 during the observation period after adding 10 µM of CpG to the samples. Lower concentrations of CpG did not induce any Ca^2+^ signal. Understandably, the Ending value was the most sensitive parameter regarding responsivity to CpG, and ending values were significantly higher compared to not-activated samples in all subsets except for ASCs (Figure 3 and Figure 4E, Supplementary Table S2).

### 2.2. B Cell Subsets Expressing IgG-Type BCR Were More Sensitive to Stimuli Than IgM-Expressing Cells

**Figure 5:**
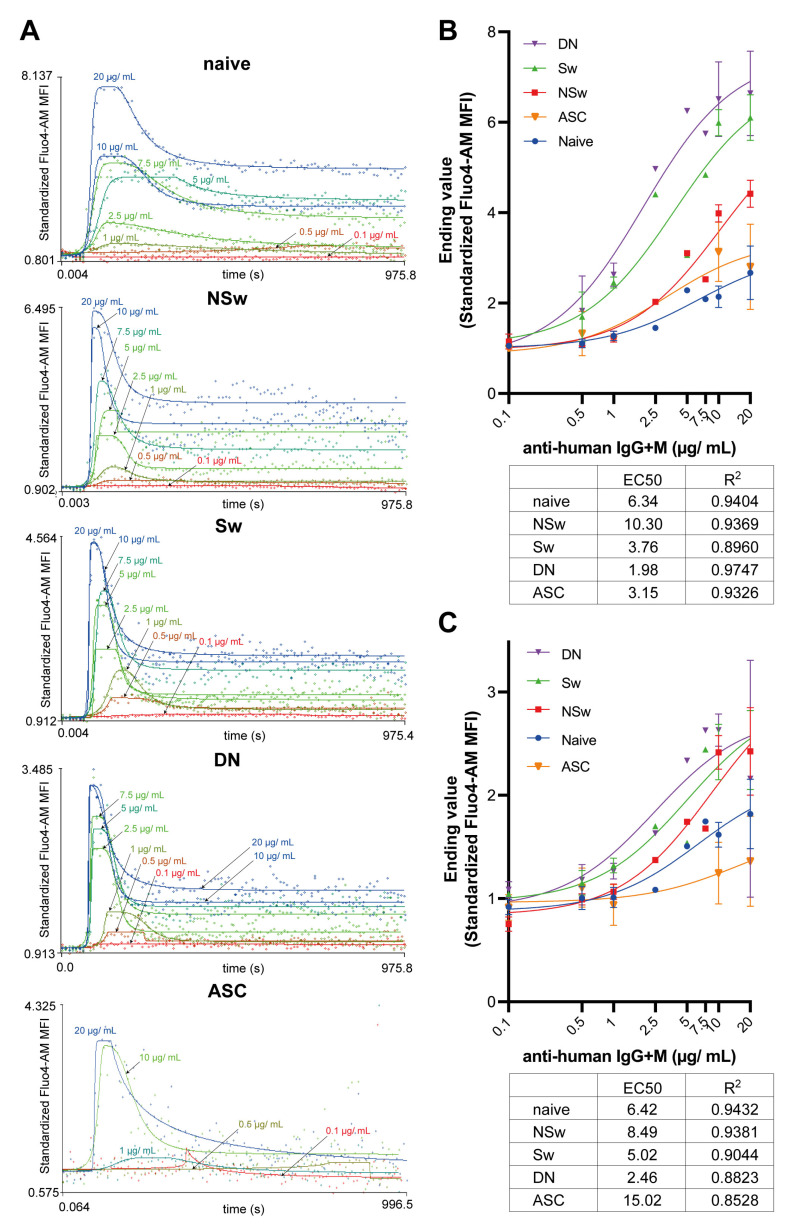
Titration of anti-human IgG+M on B-cell subsets. (**A**) Representative calcium flux kinetic curves of B-cell subsets in response to increasing concentrations of F(ab’)_2_ anti-human IgG+M. Each curve was fitted to the median of the fluorescent signal of the selected B-cell subpopulation. X-axis: time, Y-axis: standardized Fluo-4-AM median fluorescence intensity, where the baseline absolute MFI value of Fluo-4 AM is standardized to 1 and relative parameter alterations are shown. Unstimulated controls were acquired for the first 60 s; activated samples were recorded for 15 min (900 s). (**B**) Comparison of Max values reached in different B-cell subsets in response to increasing concentrations of F(ab’)_2_ anti-human IgG+M. X-axis: concentrations of anti-human IgG+M in logarithmic scale, Y-axis: standardized maximum value of Fluo-4 MFI. (**C**) Comparison of Ending values detected during the plateau phase of activation in different B-cell subsets following stimulation by increasing concentrations of F(ab’)_2_ anti-human IgG+M. X-axis: concentrations of anti-human IgG+M in logarithmic scale, Y-axis: standardized Ending value of Fluo-4 MFI. Nonlinear curve fitting was performed as a dose–response stimulation/[agonist] vs. response model in GraphPad Prism 9. R squared for goodness-of-fit of each curve and calculated EC50 values are shown in the respective table.

To compare the BCR-reactivity of B-cell subsets, we used a titration series of anti-IgG+M from 0.1 to 20 µg/mL. Sw, DN memory cells, and ASCs were more sensitive to anti-IgG+M stimulation than naive and NSw memory cells. DN and Sw memory cells responded to 0.5 µg/mL of anti-IgG+M and reached higher Max values at every concentration (Figure 5A). Their EC50 value was lower compared to other subsets (Figure 5B). Regarding the Max value, the titration curve of the NSw memory subset was between IgG-expressing memory B cells and naive B cells, and this subset reached the EC50 value at the highest concentration (Figure 5A,B). Naive cells gave the lowest response to anti-IgG+M stimulation, and their Max values were comparable to those of ASCs (Figure 5B).

The Ending value represents the plateau phase of the activation, which is the consequence of the store-operated Ca^2+^ entry (SOCE) mechanism. The plateau phase developed earlier in IgG-expressing memory subsets. DN and Sw memory cells already exhibited a plateau phase at 0.5 µg/mL of anti-IgG+M, while NSw and Naive cells first showed a plateau phase at around 2.5 µg/mL and 5 µg/mL, respectively (Figure 5A). The peak of the Ending value was similar in all memory subsets (NSw, Sw, and DN). However, the EC50 value was the lowest in the DN population, higher in Sw cells, and the highest in NSw memory cells. ASCs showed a much lower plateau phase than memory subsets (Figure 5C).

### 2.3. CD27^−^ Naive Cells Gave a Robust Calcium Flux Response to Stimulation via Their IgD Receptor That Curbed Their Responsivity to Subsequent Anti-IgD and Anti-IgM Stimulation

The cell surface IgD expression is essential to distinguish B-cell subpopulations, as it is the only way of differentiating NSw from Sw memory cells and naive cells from DN memory cells. We wanted to characterize how this labeling process impacts the responsivity of B-cell subsets to further stimulation via the BCR. IgD had to be left out of the staining to answer this question, and we could only differentiate naive and memory cells based on their CD27 expression. Technically, the CD27^−^ compartment also contains the DN memory cells. However, the average prevalence of naive cells is 12 times higher. Thus, we assumed that the kinetic curves fitted to the group’s median signal mainly represented the naive population’s response. The memory compartment contained both NSw and Sw memory cells, of which only the NSw memory cells responded to anti-IgD and anti-IgM. However, as the kinetic curves are fitted to the alteration of the median fluorescent signal of a population, the change in the MFI value generated by the response of NSw cells to anti-IgD and anti-IgM was sufficient to evaluate the effect of anti-IgD pre-treatment.

**Figure 6:**
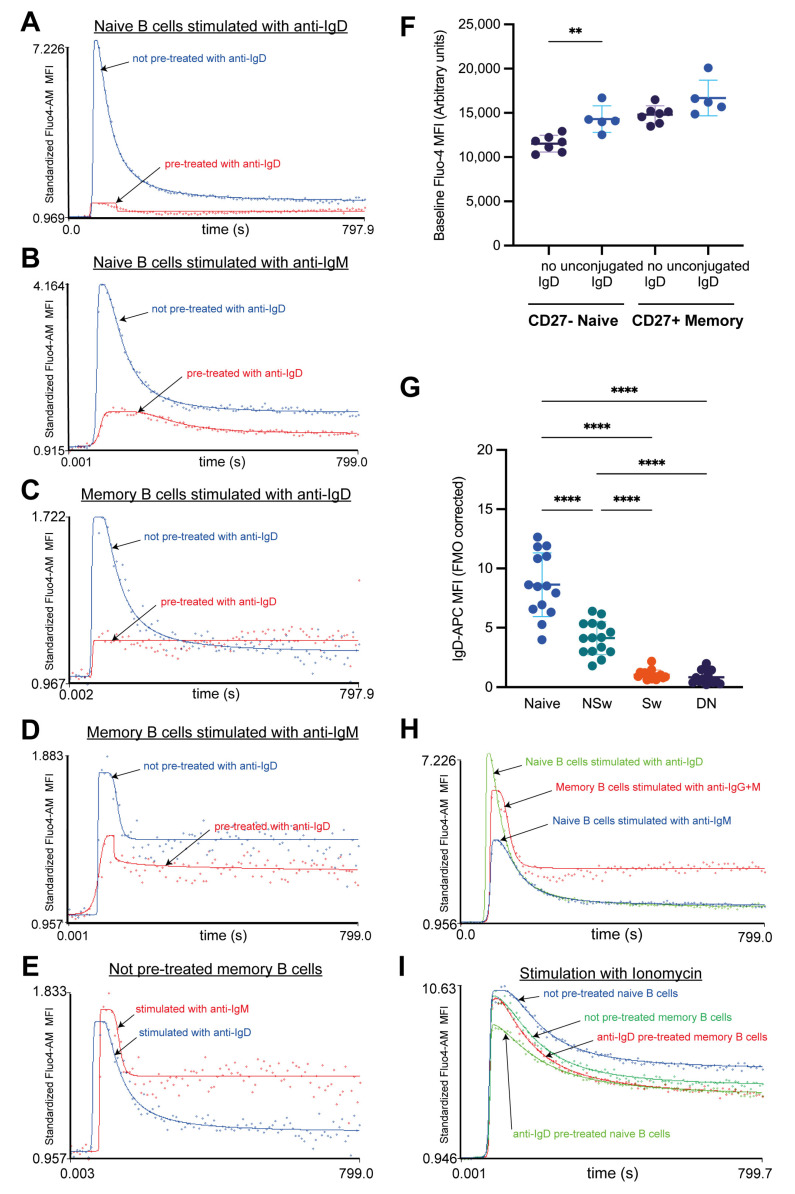
The pre-activating effect of anti-IgD labeling. Representative calcium flux kinetic curves to demonstrate the pre-activating effect of anti-IgD labeling on the (**A**) anti-IgD^−^, (**B**) anti-IgM responsivity of naive B cells; (**C**) anti-IgD^−^, (**D**) anti-IgM responsivity of CD27^+^ memory B cells. (**E**) Representative curves to compare the calcium flux kinetic pattern of CD27^+^ memory B cells (not pre-activated) to anti-IgD and anti-IgM stimulation. X-axis: time, Y-axis: standardized Fluo-4-AM median fluorescence intensity, where the baseline absolute MFI value of Fluo-4 AM is standardized to 1 and relative parameter alterations are shown. Unstimulated controls were acquired for the first 60 s for baseline, and activated samples were recorded for 12 min (720 s). (**F**) The effect of anti-IgD labeling on the baseline calcium level (represented by the median fluorescent intensity (MFI) of Fluo-4 during baseline acquisition). Each dot represents a separate measurement, all from one healthy individual, the middle line represents the mean of MFI values, and whiskers are set to the SD. (n = 7 no IgD labeling, n = 5 treated with unconjugated anti-IgD, all from one individual). Statistical comparison between the groups was performed by two-way ANOVA with Tukey’s post hoc test. (**G**) The expression of IgD in B-cell subsets was calculated by normalizing the MFI of APC to the fluorescent minus one (FMO) control (Sample-MFI/FMO-MFI) of each subset. Data are presented as mean ± SD; each dot represents the median value of all measurements from one individual, n = 16. X: B-cell subsets, Y: relative parameter value. Statistical comparison between the groups was performed by two-way ANOVA with Tukey’s post hoc test. (**H**) Representative calcium flux kinetic curves to demonstrate the responsivity of not-preactivated naive (CD27^−^) B cells to anti-IgD and anti-IgM and of not-preactivated memory (CD27^+^) B cells to anti-IgG+M. (**I**) Comparison of the responsivity of naive (CD27^−^) and memory (CD27^+^) B cells to ionomycin stimulation with and without anti-IgD pre-treatment. ** *p* < 0.01, **** *p* < 0.0001, NSw: non-switched; Sw: switched, DN: double negative memory B cells, ASC: antibody-secreting cells.

The anti-IgD pre-treatment almost wholly abolished the responsivity of CD27^−^ naive cells to anti-IgD (Figure 6A) and markedly decreased their response to anti-IgM stimulation (Figure 6B). The anti-IgD pre-treatment also significantly changed the shape of the subsequent kinetic curve of naive B cells generated by anti-IgM addition, prolonging the response time, curbing the max value, and decreasing the height of the plateau. Interestingly, the response time was not extended by the consequent IgD stimulation on naive cells, suggesting a different mechanism of action in inhibiting the two Ig receptors.

CD27^+^ memory B cells—containing both the NSw and Sw memory compartment—could also be activated by anti-IgD antibodies. The pre-treatment with anti-IgD resulted in a curbed max value but no alteration in the plateau phase (ending value) in the consequent anti-IgD stimulation in memory cells (Figure 6C). The anti-IgD pre-treatment decreased the Ca^2+^ flux response of memory cells to anti-IgM, but the pattern of the kinetic curve remained similar to the not-preactivated curve (Figure 6D).

When comparing the Ca^2+^ flux response of not-preactivated CD27^+^ memory cells to anti-IgD and anti-IgM stimulation, the Ca^2+^ flux curve after anti-IgM stimulation showed a memory-type response with a higher plateau phase. At the same time, the Ca2^+^ flux curve generated by anti-IgD stimulation resembled the pattern of naive cells with a continuously decreasing, lower plateau phase (Figure 6E). This observation further supports the different roles of IgD- and IgM-type BCR.

Unlabeled anti-IgD caused an elevation in the baseline Ca^2+^ level of CD27^−^ naive B cells but not in CD27^+^ memory cells. Despite this increase due to pre-treatment, the basal Ca^2+^ level of naive cells remained lower compared to memory B cells (Figure 6F).

Naive B cells expressed more cell surface IgD than NSw memory cells (Figure 6G). Contrary to previous beliefs, we found that when omitting anti-IgD pre-treatment, CD27^−^ naive B cells were more responsive to BCR stimulation than CD27^+^ memory B cells. CD27^−^ naive cells showed higher peak intracellular Ca^2+^ levels following anti-IgD activation than CD27^+^ memory B cells after anti-IgG+M stimulation. CD27^−^ naive cells’ IgD responsivity was two-fold higher than their IgM responsivity, and the latter remained lower compared to memory subsets (Figure 6H). The naive cells showed a different Ca^2+^ flux kinetic pattern than memory subsets; after a high peak (max value), they reached a lower plateau, showing a gradual, slow decrease until the end of the measurement period.

Stimulation with ionomycin reflects the total Ca^2+^ mobilizing capacity of the cells. Pre-treatment with anti-IgD decreased the AUC of the subsequent kinetic curve generated by ionomycin stimulation in naive cells but not memory B cells (Figure 6I). However, both naive and memory subsets responded with an intense Ca^2+^ flux to subsequent ionomycin stimulation after anti-IgD pre-treatment, indicating that the altered IgM responsivity of naive B cells was not due to the exhaustion of the Ca^2+^ stores.

## 3. Discussion

Intracellular Ca^2+^ flux acts as a central signaling pathway capable of encoding and transducing distinct BCR signaling with compelling biological and pathological consequences [2]. It is modulated by a complex interplay between various receptors and mechanisms [1,4], all contributing to a distinct Ca^2+^ flux pattern characteristic of the developmental stage and the functional status of B cells. This makes Ca^2+^ flux an ideal functional assessment for B cells. As mutations in the BCR signaling pathway, notably those that alter Ca^2+^ mobilization, are associated with autoimmune disorders and B-cell malignancies [1,2,3], Ca^2+^ flux measurements could provide further insights into disease-associated B-cell dysfunction. However, experimental design on clinical samples often necessitates tailoring protocols to match the resources available in clinical settings. 

Here we present a practical approach that allows measuring the Ca^2+^ flux characteristics of selected B-cell subpopulations by combining classical Ca^2+^ flux measurements with cell-surface staining that can be tailored to specific immunological questions. Adding cell-surface staining allows a better definition of subpopulations by gating; however, detailed panel design and considering technical artifacts are pivotal. In this publication, we used a widely accepted gating method to identify naive and various subsets of memory B cells in combination with Fluo-4-AM as a Ca^2+^ indicator. To the best of our knowledge, this is the first study where a comparative analysis of the commonly used B cell-activating agents was performed and the Ca^2+^ flux responses of circulating human B-cell subpopulations were assessed. 

Regarding experimental design, no “one size fits all” approach is available in Ca^2+^ flux measurements, as the research question, resource availability, and technical possibilities must be considered during the planning phase. The cytometer at hand largely determines the choice of Ca^2+^ indicator and the platform of post-measurement analysis. While ratiometric dyes such as Fura-2 or Indo-1 are an optimal first choice, they can only be used on cytometers equipped with a UV laser, which is not widely available. Single-wavelength dyes such as Fluo-4 AM can be a good alternative for cytometers without a UV laser; however, as they are not developed for quantitative measurements, only relative comparisons should be made on such data. As a next step, the fluorescence signals of Ca^2+^ dyes have to be normalized to allow the comparison of the fluorescent signal changes between subsets and experiment days. With ratiometric dyes, dividing the fluorescent signals of the Ca^2+^-bound and -free states is enough.

On the other hand, the fluorescent signal should be divided by the average resting fluorescence for single-wavelength dyes, making the acquisition of a baseline Ca^2+^ signal necessary [38]. This raises the following question regarding the build of the cytometer and whether the sample tube can be accessed during the acquisition. As many cytometers do not have this option, the tube must be removed after the baseline measurement to add the activating agent. Regardless of whether the sample tube is removed, the time point when the activating agent was added has to be recorded precisely for the accurate generation of kinetic parameters. This function is not available in the commonly used acquisition software. When measurement data are appended after the removal of the sample tube, the time parameter is recorded automatically and cannot be modified afterward during the analysis, leading to a distortion of the kinetic data. Compared to other kinetic analysis platforms, Facskin solves this problem by offering the option to input the precise time from activation to measurement manually. Facskin also has a built-in normalization function that can be adjusted to the measured baseline’s length, facilitating the data analysis and comparability of different subpopulations, measurement days, and activating agents.

Other than the smoothing method commonly used by kinetic analysis platforms, Facskin uses an algorithm to fit functions to the kinetic changes of the Ca^2+^ signal, making a precise mathematical description of the Ca^2+^ signal possible. The way Ca^2+^ signals are interpreted as specific cellular responses involves decoding the changes in the concentration, amplitude, steepness, and duration of the response [4]. Facskin generates parameters to describe each distinct aspect of the activation. The Max value reflects the peak of the Ca^2+^ signal, which selectively activates the nuclear factor kappa B and the c-Jun N-terminal kinase pathways [39]. The Max value is also the parameter that shows the reactivity of a given subset to an activating agent the most precisely. The Ending value in this experimental setup (12 min of observation) reflects the Ca^2+^ level during the plateau phase of the activation curve, which is a consequence of sustained extracellular Ca^2+^ entry via the CRAC channels. This lower, sustained plateau selectively stimulates the nuclear factor of the activated T cell (NFAT) pathway [1,39]. The time to reach the 1st 50% value is the most sensitive parameter to determine how fast a cell type reacts to a given activating reagent. The slope parameter at the 1st 50% closely relates to the distinct activating agent. The AUC value shows the total Ca^2+^ mobilizing ability of the given subset in response to a specific activating stimulus. 

The research question should determine the choice of activating agent, as we demonstrate here the differences in the responsivity of B-cell subsets to various activating agents. In our experiment, naive B cells responded to anti-IgM and anti-IgG+M with a smaller, slower, and more prolonged Ca^2+^ flux response than all memory subsets. NSw cells had the highest baseline Ca^2+^ level; it was higher both compared to naive and other memory B subsets. These cells gave a strong and fast Ca^2+^ flux response to anti-IgM but not anti-IgG. Despite using IgM as a BCR, NSw cells did not respond like naive cells but showed a Ca^2+^ flux kinetic pattern like the IgG-type BCR (Sw and DN) memory subsets. However, their Max value remained lower, consistent with the differences between IgM- and IgG-type BCR [40,41]. Sw and DN memory cells only responded to anti-IgG (and anti-IgG+M) despite containing approximately 10% of IgM-expressing cells. IgG-responding Sw and DN memory cells had similar basal Ca^2+^ levels, and their Ca^2+^ flux response curve pattern was almost identical. It is important to note that in both Sw and DN compartments, about half of the cells do not express IgM or IgG, likely corresponding to IgA memory cells, which should be considered in an experimental setup aiming to dissect the Ca^2+^ flux responses of B-cell memory subsets. On the other hand, when seeking to assess all large B-cell subsets parallelly, the F(ab’)_2_ fragment anti-human IgG + IgM (H+L) used in our experimental setup, can be an ideal choice, as it shows cross-reactivity to all human Ig subtypes, providing an adequate activating signal to all B-cell subsets.

Ionomycin causes rapid Ca^2+^ mobilization from the intracellular stores, followed by SOCE, and can be an ideal choice for monitoring the total Ca^2+^ mobilizing capacity of different B-cell subsets. However, the Ca^2+^ flux curve pattern after ionomycin activation differs from activation via the BCR. In our experiments, ionomycin caused a faster and several times higher peak Ca^2+^ flux and plateau in all subsets compared to anti-IgM or anti-IgG. This sustained, increased Ca^2+^ flux could be connected to the strong NFAT-activating effect of ionomycin [42]. Overall, using 1 µg/mL of ionomycin leads to a markedly stronger activation response than the BCR-induced Ca^2+^ flux and may result in more robust NFAT activation than anti-IgG+M.

Cell-surface labeling combined with Ca^2+^ flux measurements can have many advantages compared to defining cell populations by methods such as cell sorting. For instance, certain peripheral memory B-cell subsets are rare, and cell sorting would not yield enough cells for functional studies. This is especially important with clinical samples, where taking large volumes of blood from patients with autoimmunity or malignancies would be unethical. Cell surface staining also allows us to study the effect of various continuously expressed markers on Ca^2+^ flux (such as CD38 or CD25) or gate based on the expression of multiple markers. Automated gating strategies and clustering algorithms can also be utilized when increasing the number of cell-surface markers. However, cell-surface labeling can also distort the cells’ responsivity to stimuli, as demonstrated here in the case of naive B cells. 

Defining B-cell subpopulations based on their CD27 and IgD expression is the most common approach; however, in our experiments, anti-IgD-labeled naive B cells gave a much smaller, slower, and more prolonged Ca^2+^ flux response to anti-IgG+M compared to all memory subsets. This curbed response could be the consequence of the robust Ca^2+^ signal generated by the anti-IgD labeling, which not only decreased the responsivity of naive B cells to subsequent stimulation via IgD but also IgM. This anti-IgD pre-treatment seemed to have a minor effect on NSw memory cells. Surprisingly, when leaving the anti-IgD labeling out and only using CD27 to differentiate between naive and memory B cells, we found that naive cells showed a higher Ca^2+^ flux response to BCR stimulation than memory subsets. This contradicts the common hypothesis that naive B cells are less responsive to stimuli. Both with and without anti-IgD pre-treatment, naive B cells showed a distinct Ca^2+^ flux pattern compared to memory B cells. Furthermore, this naive Ca^2+^ flux pattern was conserved in the IgD-induced response of NSw memory cells, whereas their IgM-evoked response shifted to the memory pattern, resulting in a higher plateau during the SOCE. This further supports the separate role of IgD and IgM engagement in B cells and substantiates that the observed differences between naive and memory subsets are not just the consequence of the Ig-type of the BCR. 

These findings underline the need to consider the research objective carefully during experimental design. If understanding the Ca^2+^ flux characteristics of naive B cells is the primary aim, we recommend leaving IgD out of the cell-surface panel and instead using CD27, CD24, and CD38 to define subsets and anti-IgM and anti-IgD as activating agents. Such labeling cannot separate memory subsets, but naive and transitional B-cell compartments can be well defined. On the other hand, when aiming to dissect the responsivity of various B-cell memory subsets, IgD, CD27, and CD38 are appropriate markers for population definition, while anti-IgM, anti-IgG, and anti-IgA could be used as activating agents [24]. 

After selecting the activating agent, the distinct activation thresholds of functionally different B-cell subsets should also be considered, as demonstrated here with the titration of anti-IgG+M. We measured a point above which a clear plateau phase appears, indicative of the “all or nothing” phenomenon attributed to the SOCE. Above this threshold, the plateau level continued to increase dose-dependently. We observed a lower threshold for activation in IgG-expressing memory B-cell subsets (Sw and DN memory cells) compared to both IgM-expressing NSw memory cells and naive cells. This inherent difference encoded in the BCR type is connected to the function of growth factor receptor-bound protein 2 (Grb2) [41]. While Grb2 plays a role in the assembly of the inhibitory signalosome in the membrane-bound IgM-type BCR, it also has an activating and signal-amplifying effect by stabilizing the Ca^2+^ signaling scaffold in the membrane-bound IgG-type BCR [1,40]. This mechanism is presumed to increase the antigen sensitivity of the IgG-type BCR by lowering the activation threshold [40], and we confirmed this in human peripheral B-cell subsets. 

Studying the Ca^2+^ flux of B-cell subsets provides insight into their functional status, which could be an essential driving mechanism for B-cell-mediated autoimmune conditions. Here we present an optimized flow cytometric approach to characterize the Ca^2+^ flux characteristics of human peripheral B-cell subsets. Combining a gating strategy based on cell-surface staining and kinetic Ca^2+^ flux measurements within a single tube can reduce work time, necessary sample volume, and associated costs. These are critical considerations in creating the opportunity to conduct Ca^2+^ flux studies on patient samples. Standardization methods and simplifying resources increase the availability of clinical research and multi-centric studies, facilitating the investigation of rare conditions. We demonstrate that different activating agents trigger distinct Ca^2+^ flux responses, and the features of the Ca^2+^ flux curves correlate with the differentiation stage of B-cell subsets. These findings from healthy individuals’ B cells should be considered when designing experiments to investigate B-cell function in immunopathological conditions. 

## 4. Materials and Methods

### 4.1. Human Subjects

Our study was approved by the Regional Research Ethics Committee of the Medical Center, University of Pécs (RIKEB 5913/2015), and written informed consent was obtained from all participants. The study adhered to the tenets of the most recent revision of the Declaration of Helsinki. Twenty healthy adult volunteers were enrolled after signing the informed consent form (10 women and ten men). Subjects took no regular medications, had no history of illness, received no vaccines three months before enrollment, and had no infections one month before sampling. Peripheral venous blood samples were collected in six 10 mL lithium heparin tubes (for step-by-step sample preparation instructions, see protocol in Supplementary Materials).

### 4.2. Isolation of Peripheral Blood Mononuclear Cells (PBMCs)

Peripheral human blood samples were collected in lithium-heparin-treated tubes. Samples were diluted with phosphate-buffered saline (PBS) in a ratio of 1:3, and mononuclear cells were isolated using Ficoll-Paque PLUS (GE Healthcare, Chicago, IL, USA) gradient centrifugation. Cells were then washed twice and resuspended in RPMI 1640 containing 5% fetal bovine serum (FBS) (Sigma-Aldrich, St. Louis, MO, USA) for labeling.

### 4.3. Cell Surface Labeling

Freshly isolated human PBMCs were directly labeled. Acquisition and labeling times were standardized throughout the protocol. All of the used materials are summarized in Supplementary Table S1. B-cell subsets were defined as follows: CD3-PerCP (Peridinin-Chlorophyll-Protein, Sony Biotechology Inc., San Jose, CA, USA) was used for T cell exclusion, CD19-PE (Phycoerythrin, Sony Biotechology Inc., San Jose, CA, USA) as the B-cell lineage marker, CD27-PE-Cy7 (Phycoerythrin-cyanine 7, Biolegend, San Diego, CA, USA) as the memory B-cell marker, IgD-APC (Allophycocyanin, Sony Biotechology Inc., San Jose, CA, USA) to define class-switching and CD38-APC-Cy7 (Allophycocyanin-cyanine 7, Sony Biotechology Inc., San Jose, CA, USA) to define ASCs. All fluorochrome-conjugated anti-human antibodies were titrated for 10^7^ PBMCs, and staining indices were calculated to determine the best separation of each population, most negligible unspecific binding, spread, and lowest background. Due to the pre-activating effect caused by cell surface labeling (shown in the results), all samples were labeled individually and sequentially to standardize the time passed from labeling to measuring. Samples were incubated with the antibodies for 30 min at room temperature in the dark, then washed twice in RPMI (400 g, 7 min).

For determining the cell surface expression of IgM and IgG, 107 PBMCs were labeled with the following fluorochrome-conjugated anti-human antibodies: CD19-PE, CD27-PE-Cy7, IgD-APC, IgM-BV421 (Brilliant Violet 421, Biolegend, San Diego, CA, USA), and IgG-FITC (Fluorescein isothiocyanate, BD Biosciences, Franklin Lakes, NJ, USA). Zombie NIR (Biolegend, San Diego, CA, USA) was added as a viability dye before cell-surface labeling per the protocol. No Fluo-4 was added to these samples.

### 4.4. Loading PBMCs with Fluo-4 AM

The cell-permeable Fluo-4 acetoxymethyl ester is a Ca^2+^ indicator dye that exhibits increased fluorescence upon binding Ca^2+^. Briefly, 50 µg of Fluo-4 dye (Invitrogen, Thermo Fisher Scientific, Waltham, MA, USA) was dissolved in 4.56 µL of DMSO (dimethyl sulfoxide) to reach a 10 mM stock solution. Then, 1 μL of stock was dispersed with 1 µL 20% (*w*/*v*) non-ionic detergent, Pluronic F-127, and then dissolved in 98 µL RPMI (to reach 100 µM concentration). Fluo-4 concentrations were then titrated for 107 cells, and a 5 µM loading concentration was selected.

After washing, cells were resuspended in 285 µL RPMI, and 15 µL Fluo-4 was added. PBMCs were incubated for 15 min at room temperature in the dark. After washing, PBMCs were resuspended in RPMI medium supplemented with CaCl_2_ to a Ca^2+^ concentration of 1.8 mM. Samples were then kept at room temperature in the dark for 30 min to allow complete de-esterification of intracellular AM esters. Each sample was labeled individually and measured directly after the de-esterification period.

### 4.5. Flow Cytometry Measurements and the Generation of the Calcium Kinetic Curves

Flow cytometry measurements were performed using a BD FACS Canto II (3 lasers, 4-2-2 configuration, BD Biosciences, Franklin Lakes, NJ, USA). For each sample, 60 s of baseline Ca^2+^ signal were recorded, an activating agent was added, and 720 s were recorded following activation in a new file. Due to the setup of the Canto II, the tube had to be removed to add the activating agent; therefore, the time from adding the activating agent until the beginning of recording the activation curve was measured and later entered when appending the activated file to the baseline file in the FacsKin program (Figure 1A).

Compensation matrices were calculated for each measurement day using FlowJo (V10.7.1., BD Biosciences, Franklin Lakes, NJ, USA). All gates were set in FlowJo using Fluorescent Minus One (FMO) controls for each sample. Flow stability was checked by plotting the FSC (y-axis) vs. measurement time (x-axis). In the second step, singlets (y:FSC-H/x: FSC-A) and lymphocytes (y:FSC/x: SSC) were gated, and then live cells were identified as those that were successfully loaded with Fluo-4 dye (as Fluo-4^+^), using the Fluo-4 FMO to determine where the Fluo-4 negative population would be. Viability was above 95% in all measurements. B cells were defined as CD3^−^ CD19^+^ cells, and T cells as CD3^+^, CD19^−^ cells. Within the CD19^+^ population, naive B cells were defined as IgD^+^ CD27^−^, non-switched memory cells (NSw) as IgD^+^ CD27^+^, immunoglobulin class-switched memory cells (Sw) as IgD^−^ CD27^+^, CD27^−^ class-switched or double-negative (DN) memory cells as IgD^−^ CD27^−^ and circulating antibody-secreting cells (ASCs) as CD19^+^ IgD^−^ CD27^high^ CD38^high^ cells. ( Supplementary Figure S1).

After setting the gates, data from the populations of interest were exported as separate FCS files and imported into FacsKin. FacsKin performs an algorithm-based curve fitting to the median fluorescent values of each gated population, enabling the mathematical description and statistical comparison of flow cytometry acquired kinetic measurements. The most fitting function can be selected based on the physiological process’s nature, i.e., most Ca^2+^ flux curves show a double logistic course (dlogist + function). FacsKin generates an activation curve (Figure 1B) and uses the following parameters to describe each curve: Starting value, standardized to 1 to allow comparison of subsets with different baseline Ca^2+^ levels and measurements from other days and is calculated as the limit of the function at −∞ (minus infinity); maximum value (Max), which correlates with the maximum cytoplasmic level of Ca^2+^ reached during the activation, Ending value, which is the cytoplasmic Ca^2+^ level at the end of the observation period and is calculated as the limit of the function at +∞ (positive infinity); time to reach maximum value (T to Max, s), time to reach 1st 50% value (T to 50%, s), and time from 1st 50% value to maximum value (T from 50% to Max, s); Slope at 1st 50%, which is always positive, describes how much the fluorescence intensity changes in 1 s in the ascending phase of the activation (unit: int/s, where int is the unit of the vertical axis); time from maximum value to 2nd 50% value (T from Max to 2nd 50%); and Slope at 2nd 50% value, which indicates how quickly the Ca^2+^ level decreases after the peak of the activation and is always negative (unit: int/s); and the area under the curve (AUC) value, which correlates with the total capacity of cells to mobilize Ca^2+^ via a specific activation pathway. All curves were standardized to end at 780 s. These numerical data were exported and used for statistical analysis.

### 4.6. Choosing an Optimal Concentration of the Activating Agents

First, we measured a series of kinetic measurements, where no activating agent was added to monitor any changes originating from the instrumental setup. Data from these measurements (n = 5) were pooled to create reference values for each B-cell subset. Then we tested the effect of the following activating stimuli on the Ca^2+^ mobilization of B cells: anti-human IgG, anti-human IgM, anti-human IgG+M (all from Jackson ImmunoResearch Europe Ltd, Cambridge, UK), ionomycin (Sigma-Aldrich, St. Louis, MO, USA), CpG (Hycult Biotech Inc, Uden, The Netherlands), PMA (Sigma-Aldrich, St. Louis, MO, USA), IL-4 (Sigma-Aldrich, St. Louis, MO, USA), anti-human CD40 (Sony Biotechology Inc., San Jose, CA, USA), and LPS (O83, contribution of Dr. Béla Kovács from the Department of Medical Microbiology and Immunology, University of Pécs). To determine if activating agents evoke Ca^2+^ flux and, if yes, at what concentrations, we selected the concentrations based on standard laboratory practice, scientific literature, and company recommendations. The following concentrations were tested: for anti-human IgG+M 0.1, 0.5, 1, 2.5, 5, 7.5, 10, and 20 µg/mL; for anti-human IgG and anti-human IgM 0.5, 1, 10, and 20 µg/mL; for ionomycin 0.01, 0.1, 1, and 2 µg/mL; from CpG 0.05, 1, 3, and 10 µM; for PMA 5, 10, 100, and 1000 ng/mL; for IL-4 1, 5, 10, and 100 ng/mL; from anti-human CD40 0.1, 1, 10, and 50 µg/mL; for LPS 0.1, 1, 10, 100, and 300 µg/mL.

Every activating agent was titrated on 3 individual samples on 3 different days. The optimal concentrations of the tested agents were selected based on the AUC, max, and ending values. Stimulatory reagents without stimulating effects on rapid Ca^2+^ mobilization in B cells, i.e., PMA, IL-4, anti-CD40, and LPS (O83) alone, were left out of further investigations ( Supplementary Figure S2). The optimal working concentrations from each activating reagent were 10 µg/mL from anti-IgG, anti-IgM, and anti-IgG+M, 10 µM from CpG, and 1 µg/mL from ionomycin. 

### 4.7. Sample Size

The number of replicates (samples) necessary to show the differences between B-cell subsets was defined by power analysis using the AUC, Max, and Time to 50% parameters of the kinetic data and was determined to be 15. On each measurement day, the sample was collected from one individual. On every measurement day, six samples were measured in a kinetic manner: one not-activated sample as a control and five samples activated with (1) anti-IgG, (2) anti-IgM, (3) anti-IgG+M; (4) CpG, and (5) ionomycin. The cell prevalence values were checked across samples and analyzed by drawing the mean of the 6 samples of each measurement day. The sample size did not need to be altered during the study. The values were excluded if the event number in any group was too low for adequate kinetic curve fitting. Outliers were not excluded.

### 4.8. Testing the Pre-Activating Effect of IgD

We tested the pre-activating effect of the IgD labeling used for the distinction of B-cell subsets by “labeling” five tubes with unconjugated anti-human IgD (same clone as IgD-APC) at the same concentration as the APC-conjugated anti-human IgD, five tubes with APC-conjugated anti-human IgD, and five tubes left unlabeled. All tubes were measured in the above-described kinetic manner, activated with anti-IgG, anti-IgM, anti-IgG+M, anti-IgD (10 µg/mL was added to all), and ionomycin (1 µg/mL). An additional not-activated sample was measured as a control. 

### 4.9. Controls and Reproducibility

The best laboratory practices for sample preparation, flow cytometry measurements, and intracellular Ca^2+^ mobilization tracking were followed. According to the recommendations of the International Society for Advancement of Cytometry (ISAC), the labeling and measuring steps were all performed at room temperature [43]. Regarding the timeline of IgD pre-treatment, anti-IgD was added during the cell-surface labeling step. PBMCs were then incubated at room temperature for 30 min in the dark, followed by 7 min of washing. Cells were labeled with Fluo-4 AM for 15 min in the dark, at room temperature, followed by 7 min of washing. Samples were set aside for 30 min to allow complete de-esterification of intracellular Fluo-4 AM esters. Each sample was labeled individually and measured directly after the de-esterification period. After measuring 1 min of baseline, further activating agents were added, making the total time from anti-IgD pre-treatment to subsequent activation 90 min. 

Cytometer setup and tracking (CS&T) were performed every day. Compensation matrices were specifically calculated for each measurement day in FlowJo, using compensation beads (BD) for fluorochrome-conjugated antibodies and the test subjects’ PBMCs for Fluo-4. The flow rate was kept at a standard low speed. We applied several validation steps and various controls to standardize our measurements and ensure reproducibility. Populations and marker positivity were defined using FMO controls. Not-activated samples were measured and used as a reference to exclude any Fluo-4 signal alterations originating from the technical setup. For each activating agent, a sample without loading the cells with Fluo-4 (Fluo-4 FMO) was measured in a kinetic manner to exclude any alterations in the background fluorescence due to the activation process, which could resemble a Fluo-4 signal but would be an artifact. FacsKin also allows standardizing all baseline measurements to 1, and all changes can be assessed compared to this. This way, the activation of different cell subsets or individuals with differing basal Fluo-4 signal (Ca^2+^ level) can be objectively compared.

The viability of the cells was checked in several steps. In a separate tube, a viability dye (Zombie NIR) was added at the time of cell surface labeling to check the influence of the pre-analytical processes. Viability was verified by analyzing only those intact cells that could be loaded with Fluo-4. After measuring each tube, trypan blue was used to check that the activation process did not lead to rapid cell death.

### 4.10. Statistics

Statistical analysis of prevalence data, median fluorescence intensity, and Fluo-4 baseline data was performed using GraphPad Prism 9 (Graphpad Software, San Diego, CA, USA). For normality testing, Shapiro–Wilk tests were used. Non-normally distributed datasets were analyzed using Wilcoxon matched-pairs signed-rank tests for single comparisons and Friedman test with multiple comparisons FDR-corrected after Benjamini and Hochberg to compare more than two datasets. Unpaired two-tailed t-tests were used for evaluating two normally distributed datasets, and a two-way ANOVA with Tukey’s post hoc test was used to compare more than two normally distributed datasets.

To obtain the Max and Ending values from the anti-IgG+M titration series, nonlinear curve fitting was performed as a dose–response stimulation/[agonist] vs. response model in GraphPad Prism 9. Goodness-of-fit for each curve is shown in the respective table as R squared, and EC50 values were calculated.

Statistical analysis focusing on the parameters of the Ca^2+^ flux (maximum value, ending value, AUC, time to reach maximum, time to 1st 50 %, time from 1st 50% to maximum value, time from maximum value to 2nd 50%, Slope at 1st 50%, and Slope at 2nd 50%) was performed with an R software package (x64 3.5.1) [44]. For normality testing, Shapiro–Wilk tests were used with the shapiro.test function from the stats R package. Due to the non-normally distributed datasets, the wilcoxon.test and kruskal.test commands from the stats package, when appropriate, were applied. Dunn’s Kruskal–Wallis multiple comparisons were used (dunnTest function from the FSA R package [45]) to perform post hoc tests. *P* values lower than 0.05 were considered significant, and the previously mentioned dunnTest was used to calculate the adjusted *p*-values. From dunnTest, the “bh” adjustment method was used.

## Data Availability

Flow cytometry fcs data are available at: http://flowrepository.org/. Kinetic files can be sent upon request by the following contact: Anna Bajnok (bajnok.panni@gmail.com).

## Supplementary Materials

The supporting information can be downloaded at: https://www.mdpi.com/article/10.3390/ijms24109107/s1.
