# Supplementary material for: An Optimized Flow Cytometric Method to Demonstrate the Differentiation Stage-Dependent Ca2+ Flux Responses of Peripheral Human B Cells
Source: Int J Mol Sci. 2023 May 22;24(10):9107. doi: 10.3390/ijms24109107 (PMC10219102; doi:10.3390/ijms24109107)
Supplement: Supplementary file 1 [file ijms-24-09107-s001.zip › Supplementary material.pdf]

Supplementary Table S1

Comparison of the effect of different activating agents on the calcium flux kinetic parameters of each B cell subset.

The B cell subsets are **naive**, **NSw**: non-switched memory, **Sw**: switched memory, **DN**: double-negative memory, **ASC** (= Plasmablasts (PB) + Plasmacells (PC)): antibody secreting cells.Each subset was stimulated via the IgG receptor with 10 μg/ml of goat anti-human F(ab')2 fragment specific anti-IgG, via the IgM receptor with 10 μg/ml of F(ab')2 fragment goat anti-human Fc5μ fragment specific anti-IgM, via both IgG and IgM with 10 μg/ml of goat anti-human F(ab')2 fragment IgG + IgM (H+L), with 10 μM of CpG-B DNA and with 1 μg/ml ionomycin. Each parameter of the kinetic curve was compared between the various activating agents within one B cell subset. The exact p values are listed in the table under each figure, the level of significance is marked by the intensity of the color. Those parameters which cannot be calculated or are biologically irrelevant (for example T cells donot respond to anti-IgG+M) were left grey. AUC: area under the curve.

Level of significance	
p≤	0.05
p≤	0.01
p≤	0.001
p≤	0.0001

AUC

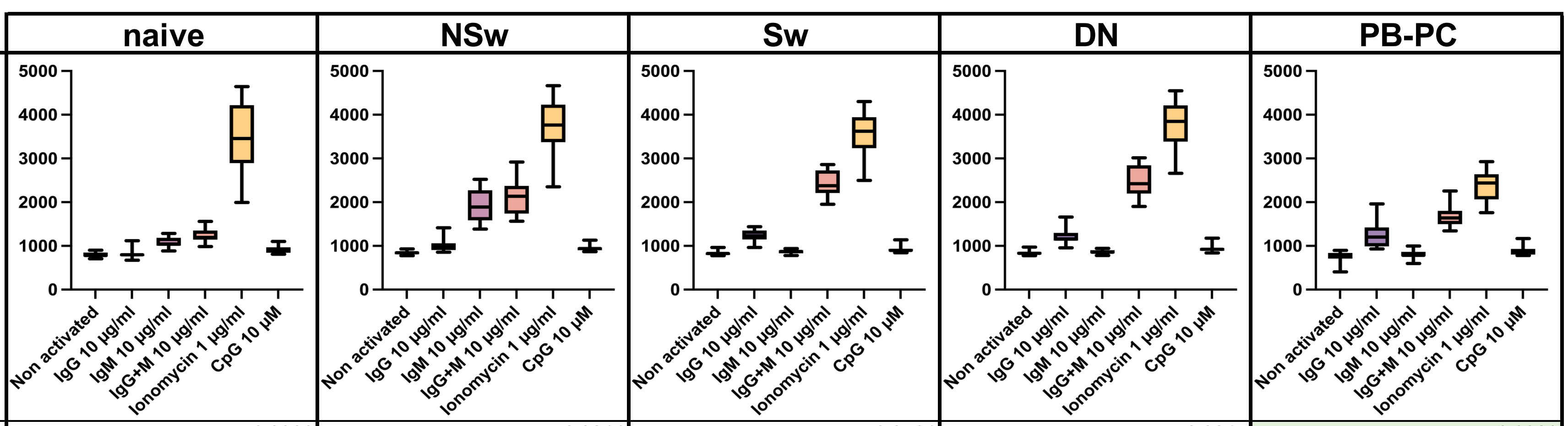

Comparison	naive	NSw	Sw	DN	PB-PC
CpG 10 uM - IgG 10 ug/ml	0.0906	0.8211	0.0561	0.0847	0.0328
CpG 10 uM - IgG+M 10 ug/ml	0.0036	0.0006	0.0004	0.0006	0.0003
CpG 10 uM - IgM 10 ug/ml	0.0931	0.0033	0.3169	0.1590	0.2721
CpG 10 uM - Ionomycin 1 ug/ml	0.0000	0.0000	0.0000	0.0000	0.0000
CpG 10 uM - Non activated	0.0576	0.0579	0.0566	0.0586	0.1446
IgG 10 ug/ml - IgG+M 10 ug/ml	0.0000	0.0012	0.1058	0.0966	0.1883
IgG 10 ug/ml - IgM 10 ug/ml	0.0007	0.0061	0.0038	0.0015	0.0012
IgG 10 ug/ml - Ionomycin 1 ug/ml	0.0000	0.0000	0.0036	0.0034	0.0119
IgG 10 ug/ml - Non activated	0.7559	0.0361	0.0001	0.0002	0.0003
IgG+M 10 ug/ml - IgM 10 ug/ml	0.2152	0.6230	0.0000	0.0000	0.0000
IgG+M 10 ug/ml - Ionomycin 1 ug/ml	0.0895	0.0619	0.1941	0.1890	0.2031
IgG+M 10 ug/ml - Non activated	0.0000	0.0000	0.0000	0.0000	0.0000
IgM 10 ug/ml - Ionomycin 1 ug/ml	0.0037	0.0202	0.0000	0.0000	0.0000
IgM 10 ug/ml - Non activated	0.0002	0.0000	0.3193	0.5623	0.6345
Ionomycin 1 ug/ml - Non activated	0.0000	0.0000	0.0000	0.0000	0.0000

Ending value

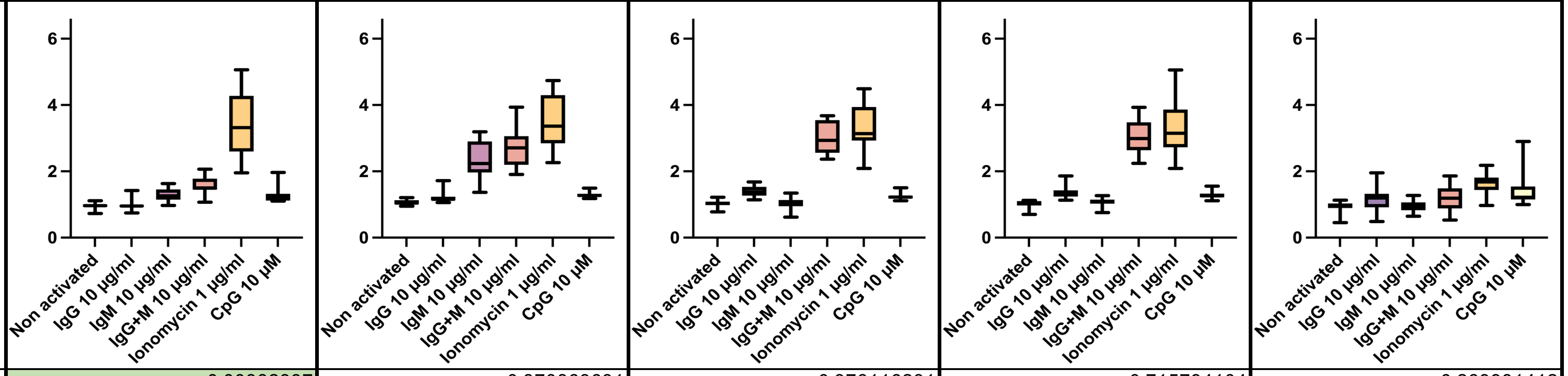

Comparison	naive	NSw	Sw	DN	PB-PC
CpG 10 uM - IgG 10 ug/ml	0.00382337	0.370863631	0.376116231	0.715794104	0.269931412
CpG 10 uM - IgG+M 10 ug/ml	0.109050634	0.001342609	0.000721822	0.001613136	0.199466757
CpG 10 uM - IgM 10 ug/ml	0.939056951	0.017472553	0.041081861	0.017794493	0.003304817
CpG 10 uM - Ionomycin 1 ug/ml	0.00054402	3.59361E-05	0.000140792	0.001395333	0.223682527
CpG 10 uM - Non activated	0.002640272	0.018757435	0.023968966	0.004026183	0.002803429
IgG 10 ug/ml - IgG+M 10 ug/ml	8.51915E-06	3.21739E-05	0.012328465	0.004910496	0.893469145
IgG 10 ug/ml - IgM 10 ug/ml	0.00282944	0.000784037	0.003641921	0.005551538	0.077929226
IgG 10 ug/ml - Ionomycin 1 ug/ml	1.47297E-10	4.8219E-07	0.003356889	0.003897581	0.043663021
IgG 10 ug/ml - Non activated	0.887606366	0.141778731	0.001690414	0.001337972	0.070344446
IgG+M 10 ug/ml - IgM 10 ug/ml	0.138489971	0.401760904	4.60267E-08	3.11253E-08	0.086470811
IgG+M 10 ug/ml - Ionomycin 1 ug/ml	0.053297457	0.317411409	0.703044108	0.850015339	0.024620278
IgG+M 10 ug/ml - Non activated	5.6098E-06	1.62277E-08	9.88938E-09	1.14043E-09	0.07635014
IgM 10 ug/ml - Ionomycin 1 ug/ml	0.000823771	0.078426043	6.4541E-09	2.43847E-08	0.000122269
IgM 10 ug/ml - Non activated	0.001968887	9.9503E-07	0.841961185	0.688079537	0.94894446
Ionomycin 1 ug/ml - Non activated	1.12685E-10	1.04219E-10	1.73613E-09	1.36653E-09	0.000141521

Max value

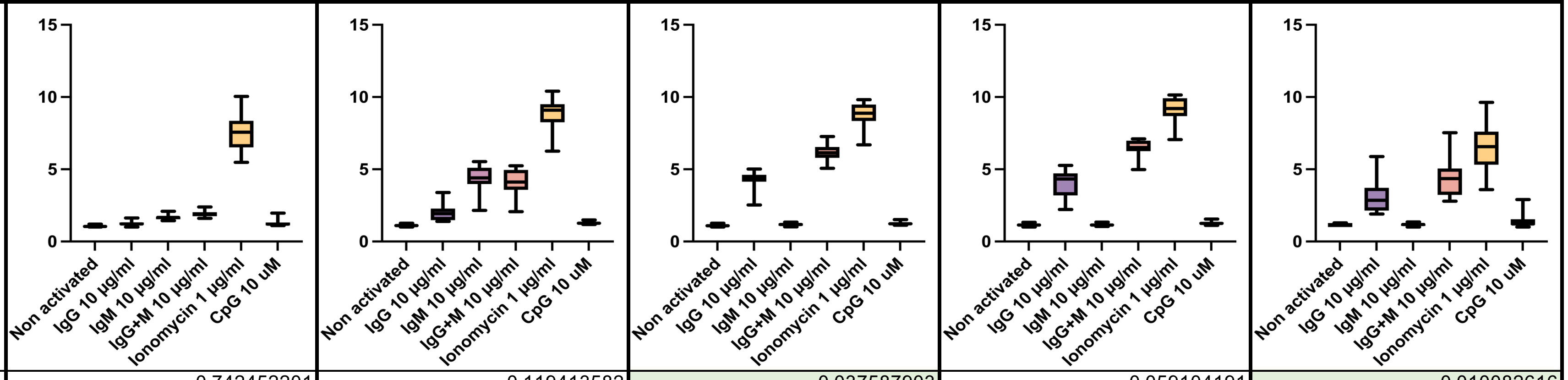

Comparison	naive	NSw	Sw	DN	PB-PC
CpG 10 uM - IgG 10 ug/ml	0.742452201	0.119413582	0.037587903	0.059104191	0.010082616
CpG 10 uM - IgG+M 10 ug/ml	0.000554222	0.000456158	0.000226521	0.000562003	8.67524E-05
CpG 10 uM - IgM 10 ug/ml	0.030108898	0.000114835	0.412577388	0.142415035	0.557343292
CpG 10 uM - Ionomycin 1 ug/ml	3.4217E-07	5.91425E-09	4.87472E-07	1.16279E-06	4.11297E-07
CpG 10 uM - Non activated	0.055597245	0.164698911	0.073771497	0.089506629	0.429693408
IgG 10 ug/ml - IgG+M 10 ug/ml	0.000170729	0.043379369	0.127142617	0.139788682	0.215704549
IgG 10 ug/ml - IgM 10 ug/ml	0.013273194	0.019130879	0.00420046	0.000595498	0.006216207
IgG 10 ug/ml - Ionomycin 1 ug/ml	7.29823E-08	1.12844E-05	0.003916244	0.003803586	0.010450084
IgG 10 ug/ml - Non activated	0.099156062	0.003522845	7.73143E-05	0.000239647	0.001774061
IgG+M 10 ug/ml - IgM 10 ug/ml	0.197133918	0.704861346	1.10214E-05	7.86393E-07	9.03197E-05
IgG+M 10 ug/ml - Ionomycin 1 ug/ml	0.099429504	0.018001634	0.163593308	0.146388794	0.218150237
IgG+M 10 ug/ml - Non activated	9.01719E-08	6.68992E-07	3.25421E-08	1.99992E-07	1.35593E-05
IgM 10 ug/ml - Ionomycin 1 ug/ml	0.004166759	0.040211562	1.51935E-08	3.32757E-10	9.46298E-07
IgM 10 ug/ml - Non activated	3.71315E-05	1.07603E-07	0.303419738	0.763920443	0.861721103
Ionomycin 1 ug/ml - Non activated	2.89378E-12	2.40845E-13	1.11352E-11	8.74034E-11	1.24737E-07

Time to 1st 50% (sec)

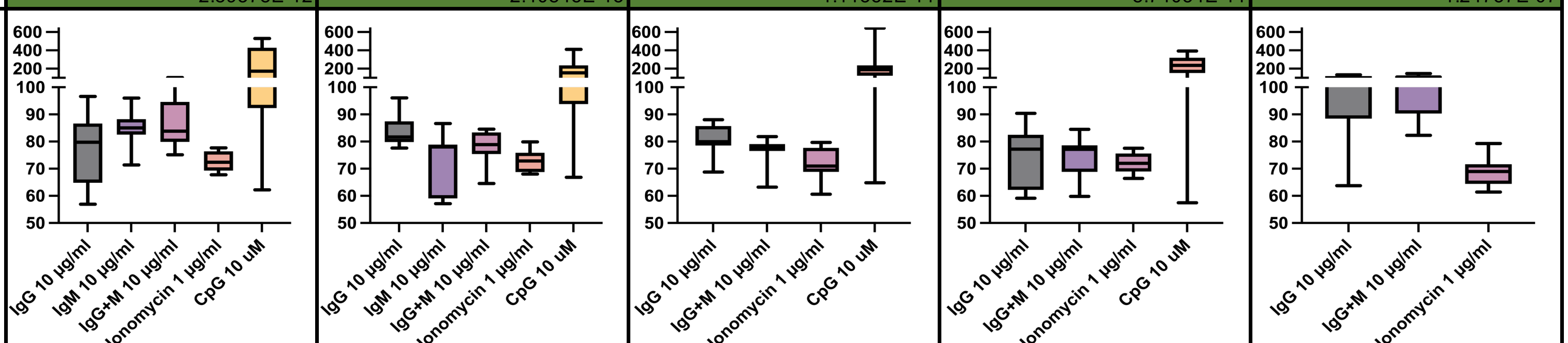

Comparison	naive	NSw	Sw	DN	PB-PC
CpG 10 uM - IgG 10 ug/ml	0.00180182	0.216374762	0.017630532	0.000397724	
CpG 10 uM - IgG+M 10 ug/ml	0.093657016	0.00562049	7.91686E-05	0.000462542	
CpG 10 uM - IgM 10 ug/ml	0.09440172	1.07904E-05			
CpG 10 uM - Ionomycin 1 ug/ml	1.26074E-06	4.84154E-06	1.05159E-07	1.65281E-05	
IgG 10 ug/ml - IgG+M 10 ug/ml	0.096338242	0.106561884	0.110822013	0.949145976	0.561340436
IgG 10 ug/ml - IgM 10 ug/ml	0.097322445	0.001153367			
IgG 10 ug/ml - Ionomycin 1 ug/ml	0.182898427	0.000441553	0.003674636	0.452712882	0.000274651
IgG+M 10 ug/ml - IgM 10 ug/ml	0.884233188	0.106961203			
IgG+M 10 ug/ml - Ionomycin 1 ug/ml	0.001367598	0.055182389	0.1520806	0.398519965	3.94659E-05
IgM 10 ug/ml - Ionomycin 1 ug/ml	0.001745345	0.703580425			

Slope at 1st 50%

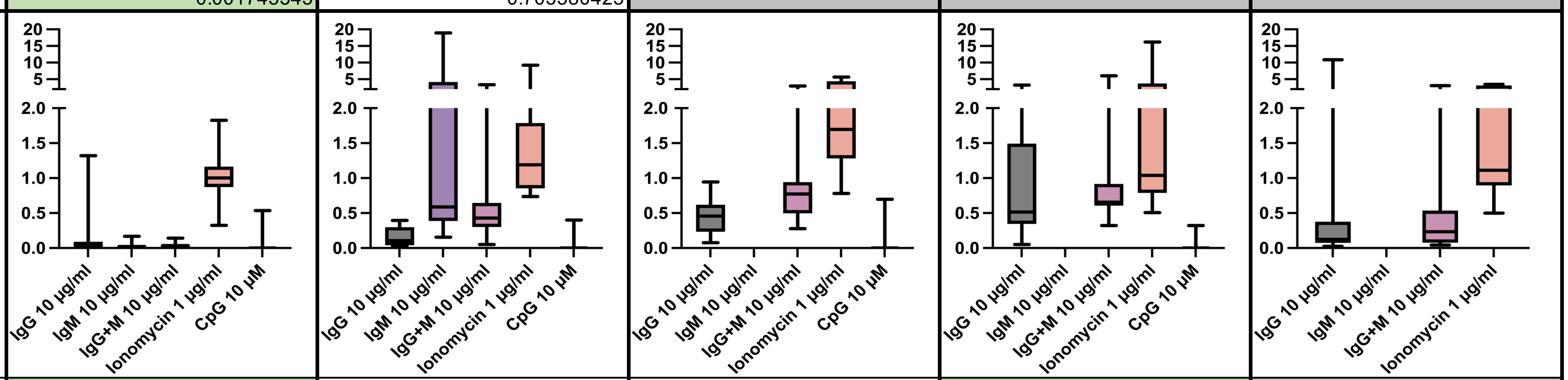

Comparison	naive	NSw	Sw	DN	PB-PC
CpG 10 uM - IgG 10 ug/ml	0.000795398	0.079000264	0.015146419	0.000314877	
CpG 10 uM - IgG+M 10 ug/ml	0.000758518	0.000115976	6.35569E-05	3.15579E-05	
CpG 10 uM - IgM 10 ug/ml	0.007688167	1.44844E-06			
CpG 10 uM - Ionomycin 1 ug/ml	1.22616E-10	1.90766E-09	4.65879E-10	5.92089E-08	
IgG 10 ug/ml - IgG+M 10 ug/ml	0.961829135	0.034517732	0.093045761	0.530540431	0.793519748
IgG 10 ug/ml - IgM 10 ug/ml	0.448247715	0.00173583			
IgG 10 ug/ml - Ionomycin 1 ug/ml	0.004546682	1.28105E-05	0.000139367	0.065125807	0.000722642
IgG+M 10 ug/ml - IgM 10 ug/ml	0.407233488	0.282089261			
IgG+M 10 ug/ml - Ionomycin 1 ug/ml	0.003210749	0.027001056	0.023956374	0.192596695	0.000940894
IgM 10 ug/ml - Ionomycin 1 ug/ml	0.000264701	0.197600435			

Time from 1st 50% to Max (sec)

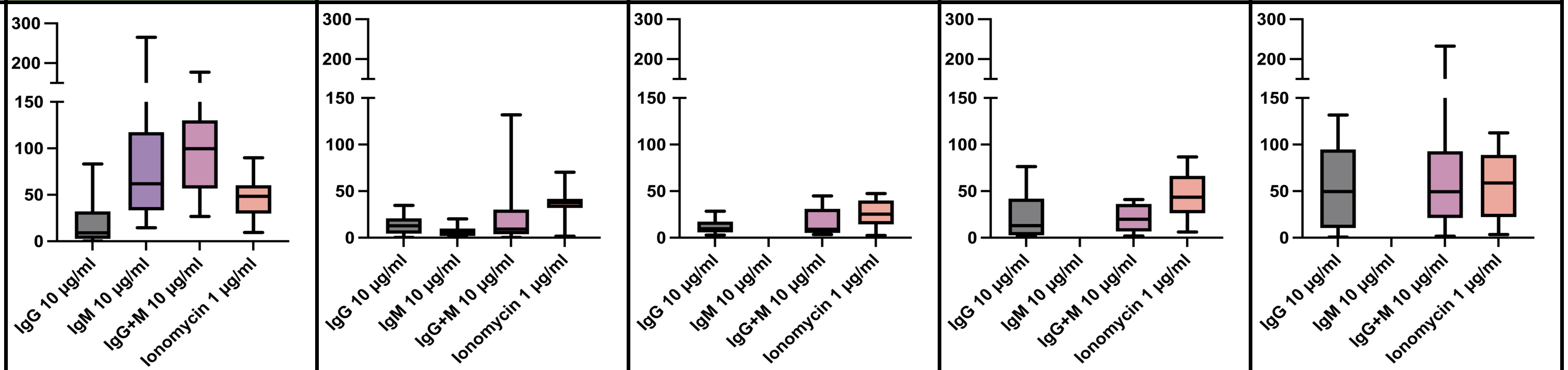

Comparison	naive	NSw	Sw	DN	PB-PC
IgG 10 ug/ml - IgG+M 10 ug/ml	0.00012402	0.915345283	0.559327722	0.639039918	0.976800518
IgG 10 ug/ml - IgM 10 ug/ml	0.003924136	0.141778205			
IgG 10 ug/ml - Ionomycin 1 ug/ml	0.077222958	0.009790607	0.037030081	0.016495669	1
IgG+M 10 ug/ml - IgM 10 ug/ml	0.236932629	0.142708425			
IgG+M 10 ug/ml - Ionomycin 1 ug/ml	0.027589633	0.009099105	0.082603275	0.030887236	1
IgM 10 ug/ml - Ionomycin 1 ug/ml	0.223412951	4.53826E-05			

Time from Max to 2nd 50% (sec)

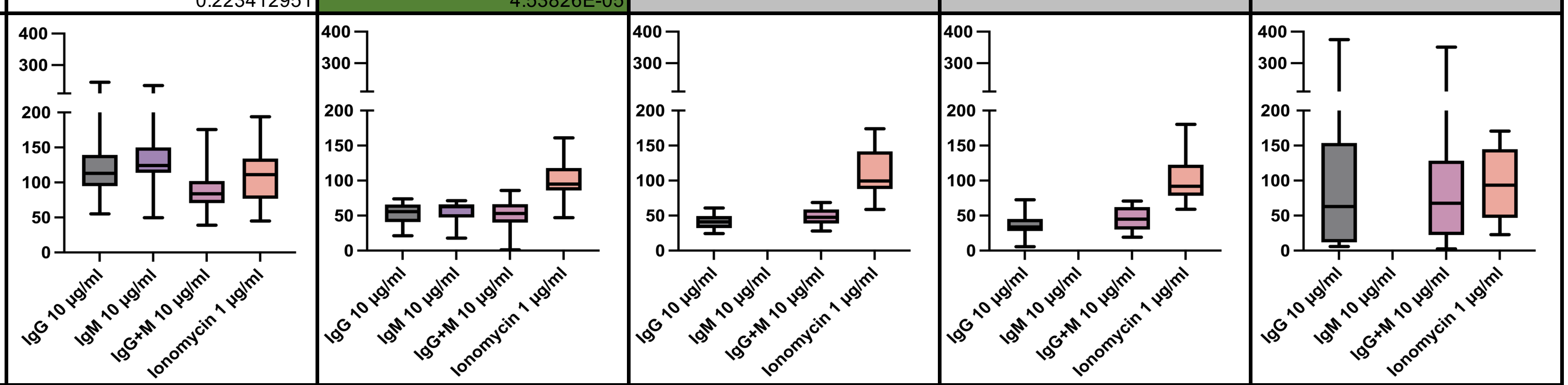

Comparison	naive	NSw	Sw	DN	PB-PC
IgG 10 ug/ml - IgG+M 10 ug/ml	0.053297429	0.837243675	0.2543368	0.340946616	0.918930366
IgG 10 ug/ml - IgM 10 ug/ml	0.450416112	0.865505279			
IgG+M 10 ug/ml - IgM 10 ug/ml	0.010661606				
IgG 10 ug/ml - Ionomycin 1 ug/ml	0.533857116	0.000196705	7.51783E-07	2.28405E-06	0.669092868
IgG+M 10 ug/ml - Ionomycin 1 ug/ml	0.235045027	0.000167545			
IgM 10 ug/ml - Ionomycin 1 ug/ml	0.19826565	0.00052855	8.8274E-05	9.13527E-05	1
IgG+M 10 ug/ml - IgM 10 ug/ml		0.860966341			

Slope at 2nd 50%

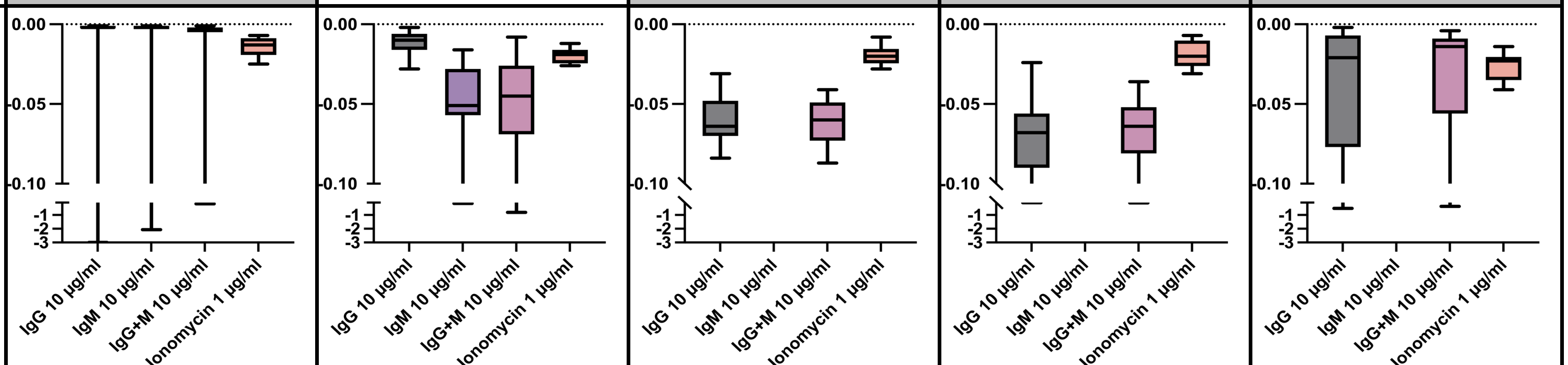

Comparison	naive	NSw	Sw	DN	PB-PC
IgG 10 ug/ml - IgG+M 10 ug/ml	0.0544371	1.46507E-05	0.886938693	0.898179824	0.853853957
IgG 10 ug/ml - IgM 10 ug/ml	0.503288423	6.43057E-06	8.59985E-06	1.50997E-05	1
IgG 10 ug/ml - Ionomycin 1 ug/ml	3.724E-05	0.08724713			
IgG+M 10 ug/ml - IgM 10 ug/ml	0.185223661	0.757889135			
IgG+M 10 ug/ml - Ionomycin 1 ug/ml	0.027633666	0.013627901	8.42029E-06	1.36412E-05	1
IgM 10 ug/ml - Ionomycin 1 ug/ml	0.000338023	0.007324678			

Time to Max (sec)

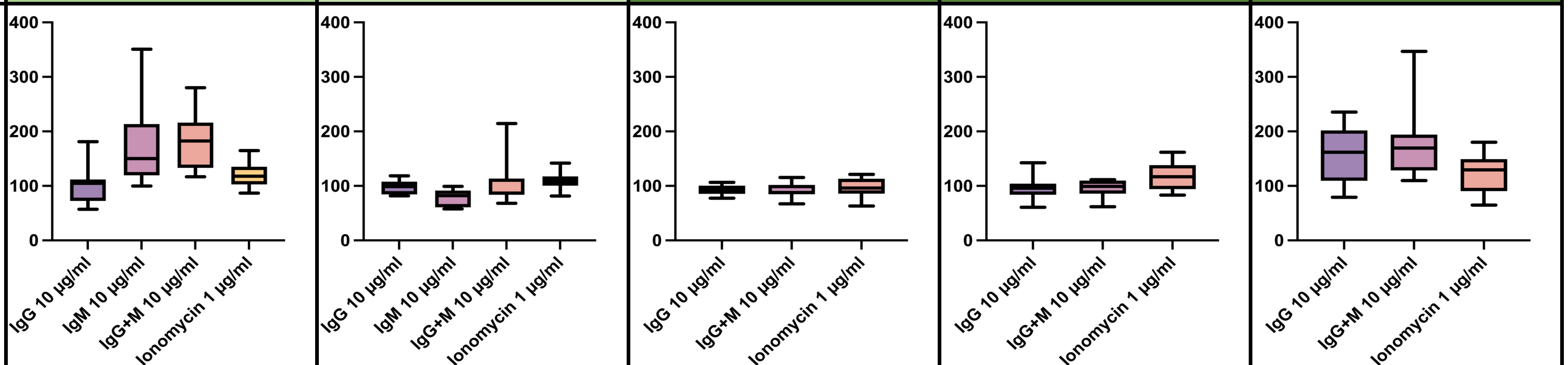

Comparison	naive	NSw	Sw	DN	PB-PC
IgG 10 ug/ml - IgG+M 10 ug/ml	0.000462248	0.496306723	0.80241931	0.864569302	0.570668962
IgG 10 ug/ml - IgM 10 ug/ml	0.005114832	0.008750584			
IgG 10 ug/ml - Ionomycin 1 ug/ml	0.359611349	0.168983838	0.445719796	0.100796526	0.072485775
IgG+M 10 ug/ml - IgM 10 ug/ml	0.357227892	0.043345577			
IgG+M 10 ug/ml - Ionomycin 1 ug/ml	0.004711371	0.048383884	0.588251757	0.07547547	0.035111703
IgM 10 ug/ml - Ionomycin 1 ug/ml	0.044559659	6.575E-05			

Supplementary Table S2

Comparison of the calcium flux kinetic response of different B cell subsets to each activating agent.

The B cell subsets are **naive**, **NSw**: non-switched memory, **Sw**: switched memory, **DN**: double-negative memory, **ASC** (= Plasmablasts (PB) + Plasmacells (PC)): antibody secreting cells. Each subset was stimulated via the IgG receptor with 10 µg/ml of goat anti-human F(ab')2 fragment specific anti-IgG, via the IgM receptor with 10 µg/ml of F(ab')2 fragment goat anti-human Fc5µ fragment specific anti-IgM, via both IgGand IgM with goat 10 µg/ml of anti-human F(ab')2 fragment IgG + IgM (H+L), with 10 µM of CpG-B DNA and with 1 µg/ml ionomycin. Each parameter of the kinetic curve was compared between the different B cell subsets within one activating agent. The exact p values are listed in the table under each figure, the level of significance is marked by the intensity of the color. Those parameters which cannot be calculated or are biologically irrelevant (for example T cells do not respond to anti-IgG+M) were left grey. AUC: area under the curve.

Level of significance	
$p \leq$	0.05
$p \leq$	0.01
$p \leq$	0.001
$p \leq$	0.0001

AUC

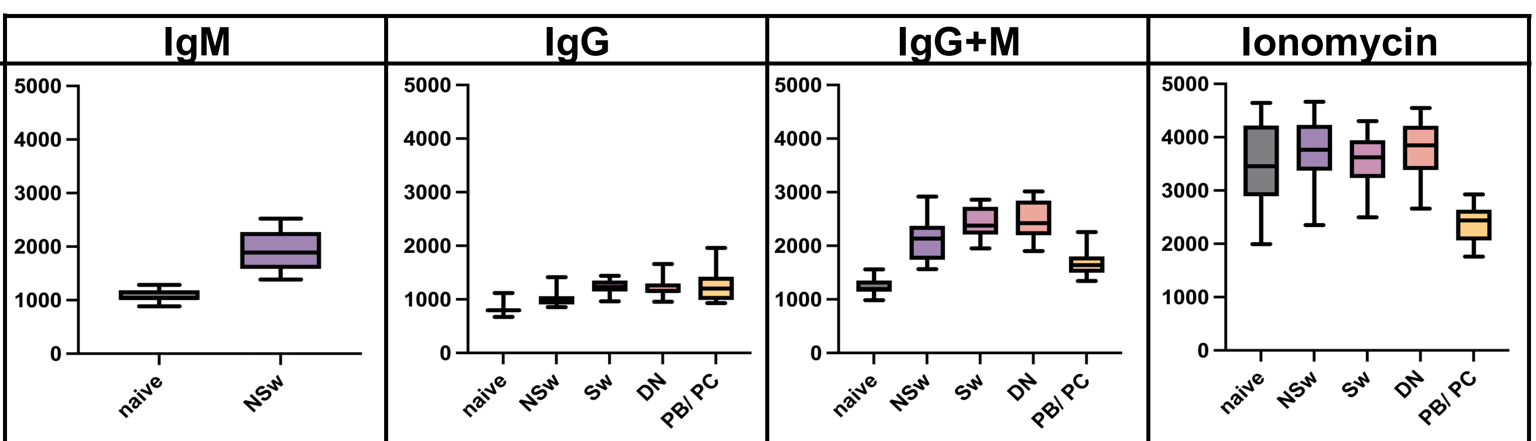

Comparison	IgM	IgG	IgG+M	Ionomycin
DN - Naiv			9.1459E-09	0.441479276
DN - NSw		0.006470024	0.080151848	0.808269365
DN - PB_PC		0.836856774	0.000125601	9.96212E-05
DN - Sw		0.806314155	0.743890225	0.444017053
Naiv - NSw	1.28935E-08		6.44862E-05	0.54430217
Naiv - PB_PC			0.0550473	0.002972862
Naiv - Sw			3.37683E-08	0.893299342
NSw - PB_PC		0.005821707	0.045971099	0.000222159
NSw - Sw		0.002216588	0.141502059	0.561402548
PB_PC - Sw		0.827081309	0.000386317	0.002078038

Ending value

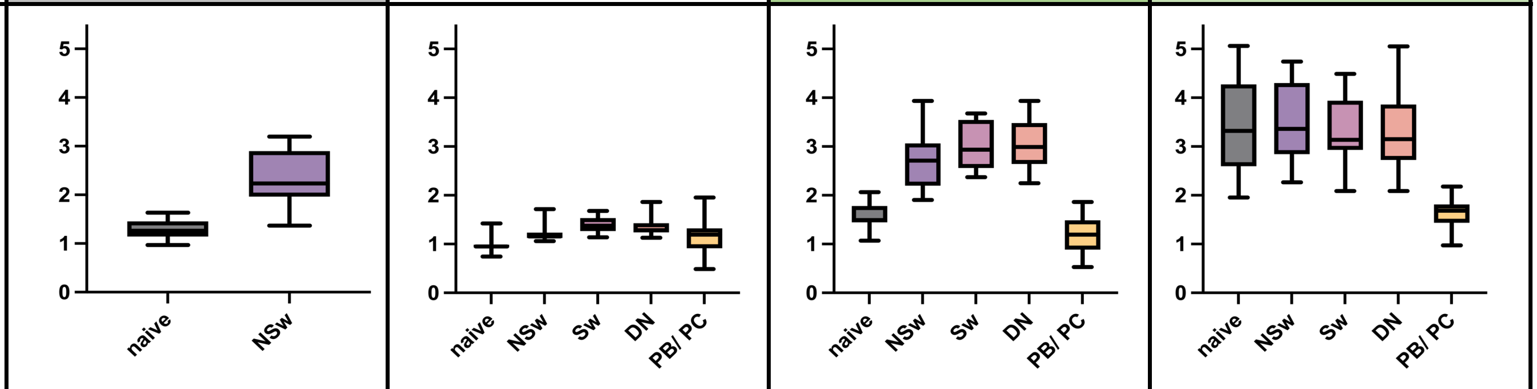

Comparison	IgM	IgG	IgG+M	Ionomycin
DN - Naiv			2.25053E-05	0.969074373
DN - NSw		0.058697205	0.520576078	0.742244358
DN - PB_PC		0.046435411	2.92905E-07	0.000407959
DN - Sw		0.738131305	0.926580564	0.929821346
Naiv - NSw	3.86804E-07		0.000477381	0.770111091
Naiv - PB_PC			0.384034591	0.000278026
Naiv - Sw			2.75141E-05	0.932702899
NSw - PB_PC		0.953662267	7.3724E-06	5.89632E-05
NSw - Sw		0.043967119	0.523623412	0.799150127
PB_PC - Sw		0.024715946	2.4697E-07	0.000201511

Max value

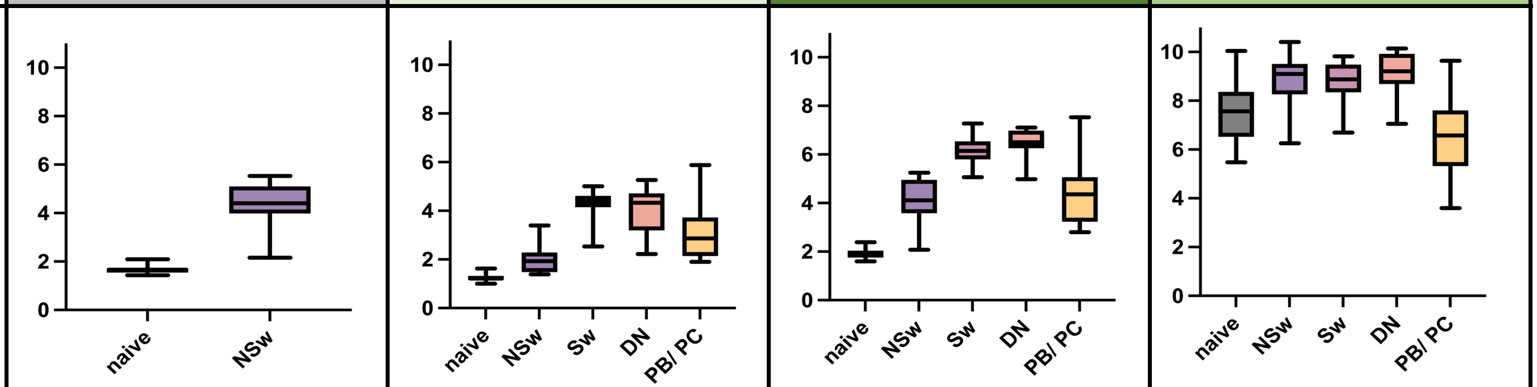

Comparison	IgM	IgG	IgG+M	Ionomycin
DN - Naiv			5.72009E-10	0.014038907
DN - NSw		6.38229E-06	0.000602382	0.653408092
DN - PB_PC		0.025501774	0.002426864	0.000663466
DN - Sw		0.782258611	0.580340681	0.460422052
Naiv - NSw	1.28935E-08		0.007158674	0.051789794
Naiv - PB_PC			0.001895738	0.29085441
Naiv - Sw			9.98887E-09	0.102511986
NSw - PB_PC		0.027719242	0.638463797	0.002725991
NSw - Sw		3.14411E-06	0.002829088	0.720378734
PB_PC - Sw		0.015709422	0.010660477	0.006315976

Time to 1st 50% (sec)

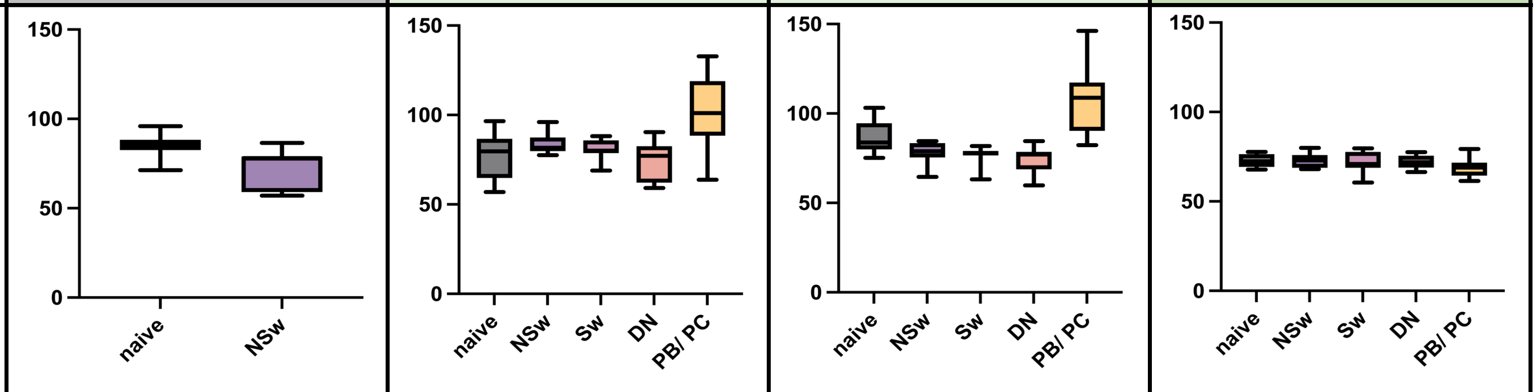

Comparison	IgM	IgG	IgG+M	Ionomycin
DN - Naiv			0.000693196	0.954928897
DN - NSw		0.048450927	0.193020718	1
DN - PB_PC		0.000144316	1.6408E-07	0.375487994
DN - Sw		0.31257262	0.574617418	1
Naiv - NSw	5.61897E-05		0.044996446	1
Naiv - PB_PC			0.063397753	0.666289339
Naiv - Sw			0.004218703	1
NSw - PB_PC		0.066778652	8.06977E-05	0.259866257
NSw - Sw		0.256333777	0.431362183	1
PB_PC - Sw		0.003697698	1.83923E-06	0.27186316

Slope at 1st 50%

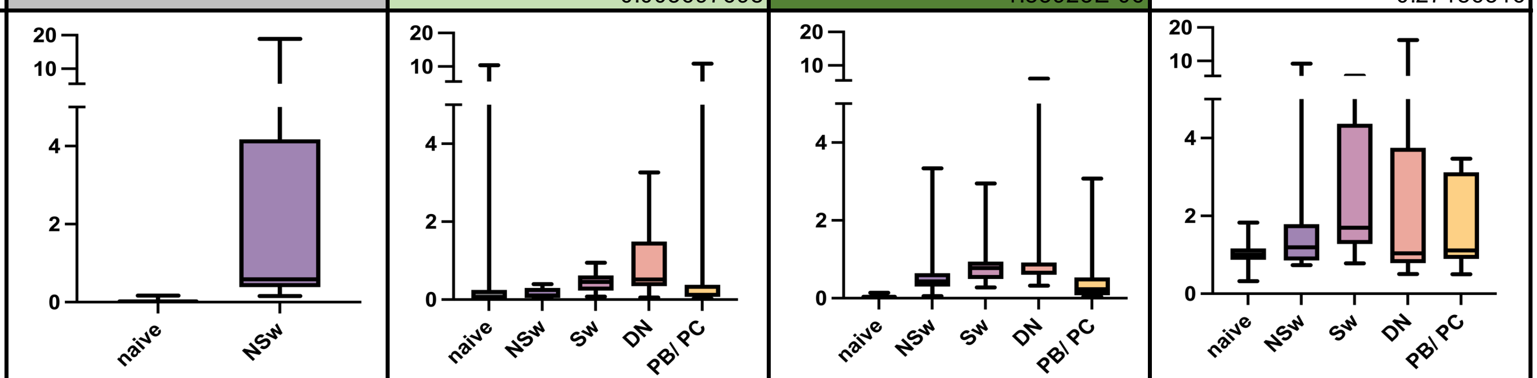

Comparison	IgM	IgG	IgG+M	Ionomycin
DN - Naiv			5.65575E-08	0.480911578
DN - NSw		0.000924056	0.119477595	0.924951134
DN - PB_PC		0.013129254	0.007653943	0.816116405
DN - Sw		0.439149692	0.919922477	0.255929539
Naiv - NSw	4.12287E-06		0.000139722	0.485687508
Naiv - PB_PC			0.008336389	0.327472639
Naiv - Sw			5.1383E-08	0.036056587
NSw - PB_PC		0.343515991	0.242369369	0.800956428
NSw - Sw		0.007814653	0.129023294	0.211645429
PB_PC - Sw		0.077747505	0.007039443	0.358175527

Time from 1st 50% to Max (sec)

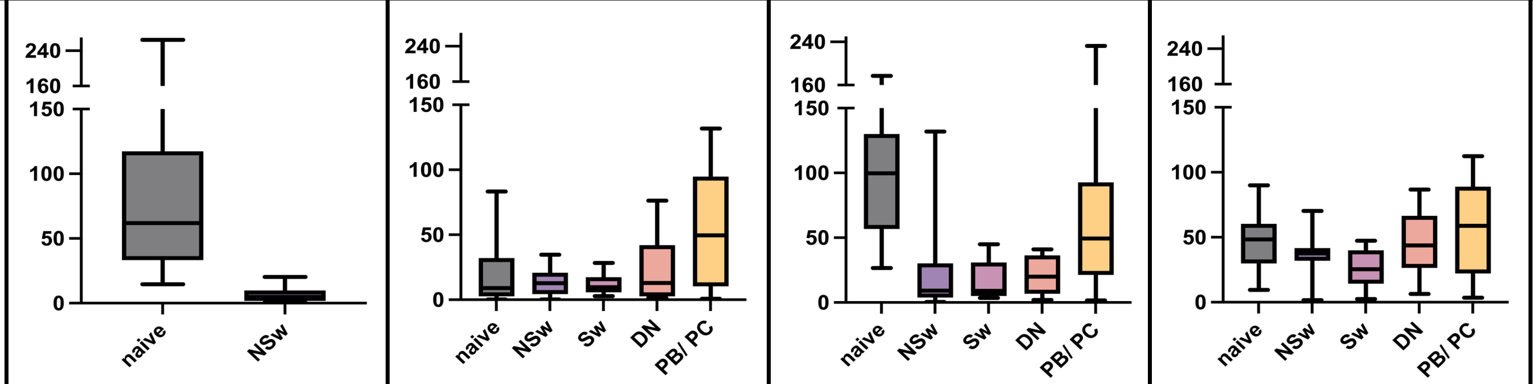

Comparison	IgM	IgG	IgG+M	Ionomycin
DN - Naiv			0.000434247	0.717234003
DN - NSw		0.916739498	0.69160205	0.76192029
DN - PB_PC		0.028041654	0.043552028	0.642065968
DN - Sw		0.934772762	0.607925485	0.111930031
Naiv - NSw	1.54721E-07		8.17127E-05	0.637868536
Naiv - PB_PC			0.144183364	0.764951651
Naiv - Sw			6.48357E-05	0.063255935
NSw - PB_PC		0.031286988	0.013189313	0.417125794
NSw - Sw		0.838681404	0.83854992	0.270891146
PB_PC - Sw		0.019185555	0.008739633	0.043334029

Time from Max to 2nd 50% (sec)

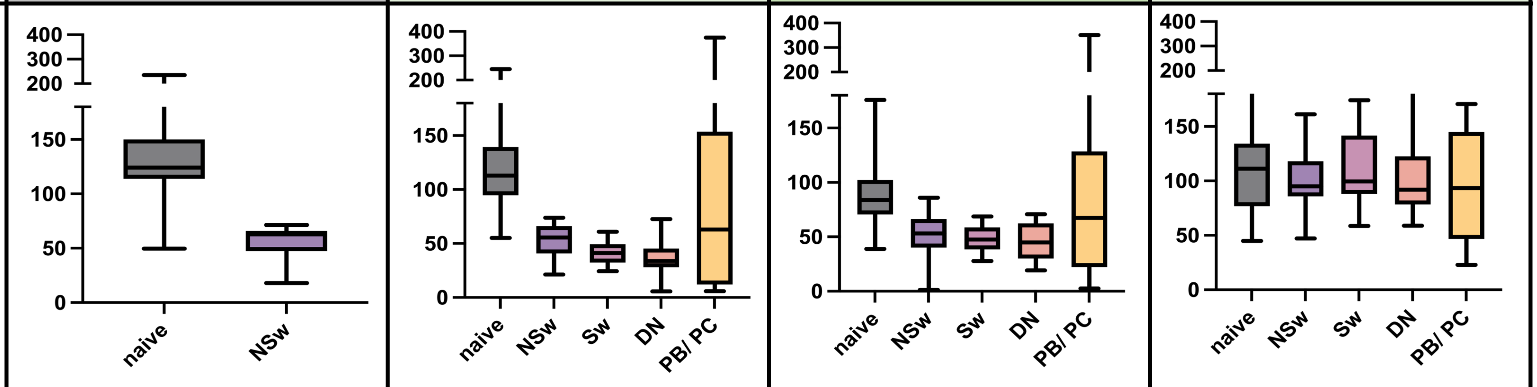

Comparison	IgM	IgG	IgG+M	Ionomycin
DN - Naiv			0.00094726	1
DN - NSw		0.084124962	0.615166106	0.964281806
DN - PB_PC		0.077894434	0.157091702	0.886914304
DN - Sw		0.557154842	0.80551144	1
Naiv - NSw	2.51422E-06		0.004320994	1
Naiv - PB_PC			0.079975709	1
Naiv - Sw			0.001912438	1
NSw - PB_PC		0.818098231	0.405143673	1
NSw - Sw		0.169077425	0.737562381	1
PB_PC - Sw		0.202392824	0.265539812	1

Slope at 2nd 50%

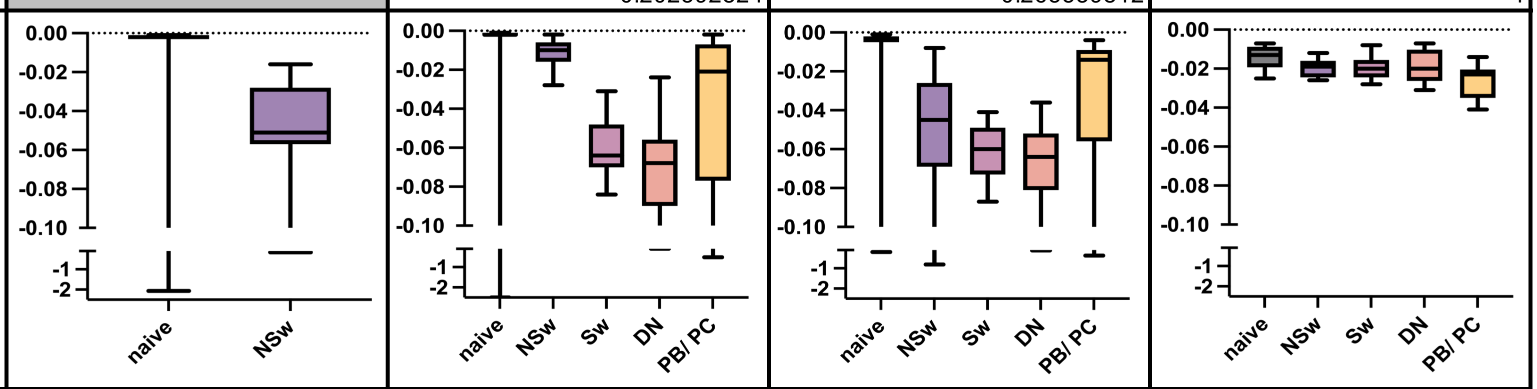

Comparison	IgM	IgG	IgG+M	Ionomycin
DN - Naiv			7.51455E-05	0.16602509
DN - NSw		1.00499E-05	0.311510162	1
DN - PB_PC		0.012107074	0.047425517	0.156186909
DN - Sw		0.561683083	0.715495227	0.918802699
Naiv - NSw	0.000644345		0.003889569	0.139731517
Naiv - PB_PC			0.065939448	0.007098029
Naiv - Sw			0.000194362	0.199735111
NSw - PB_PC		0.049111949	0.331381817	0.180385731
NSw - Sw		7.70203E-05	0.428687496	0.98530059
PB_PC - Sw		0.045636242	0.079201103	0.194635613

Time to Max (sec)

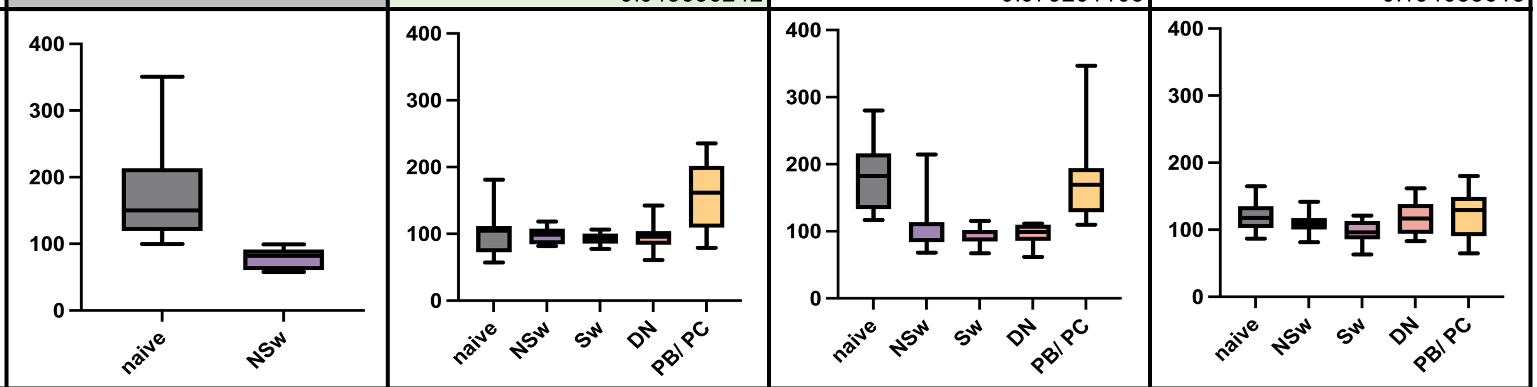

Comparison	IgM	IgG	IgG+M	Ionomycin
DN - Naiv			5.43286E-05	0.778254826
DN - NSw		0.638042452	0.993226419	0.743624795
DN - PB_PC		0.006346374	9.54557E-05	0.862103661
DN - Sw		0.765650942	0.785568344	0.142254172
Naiv - NSw	1.28935E-08		4.23115E-05	0.596543529
Naiv - PB_PC			0.836732777	0.970923502
Naiv - Sw			1.70264E-05	0.114896891
NSw - PB_PC		0.018477663	8.24934E-05	0.695883673
NSw - Sw		0.520153325	0.889202636	0.278236229
PB_PC - Sw		0.002364965	2.67737E-05	0.059404376

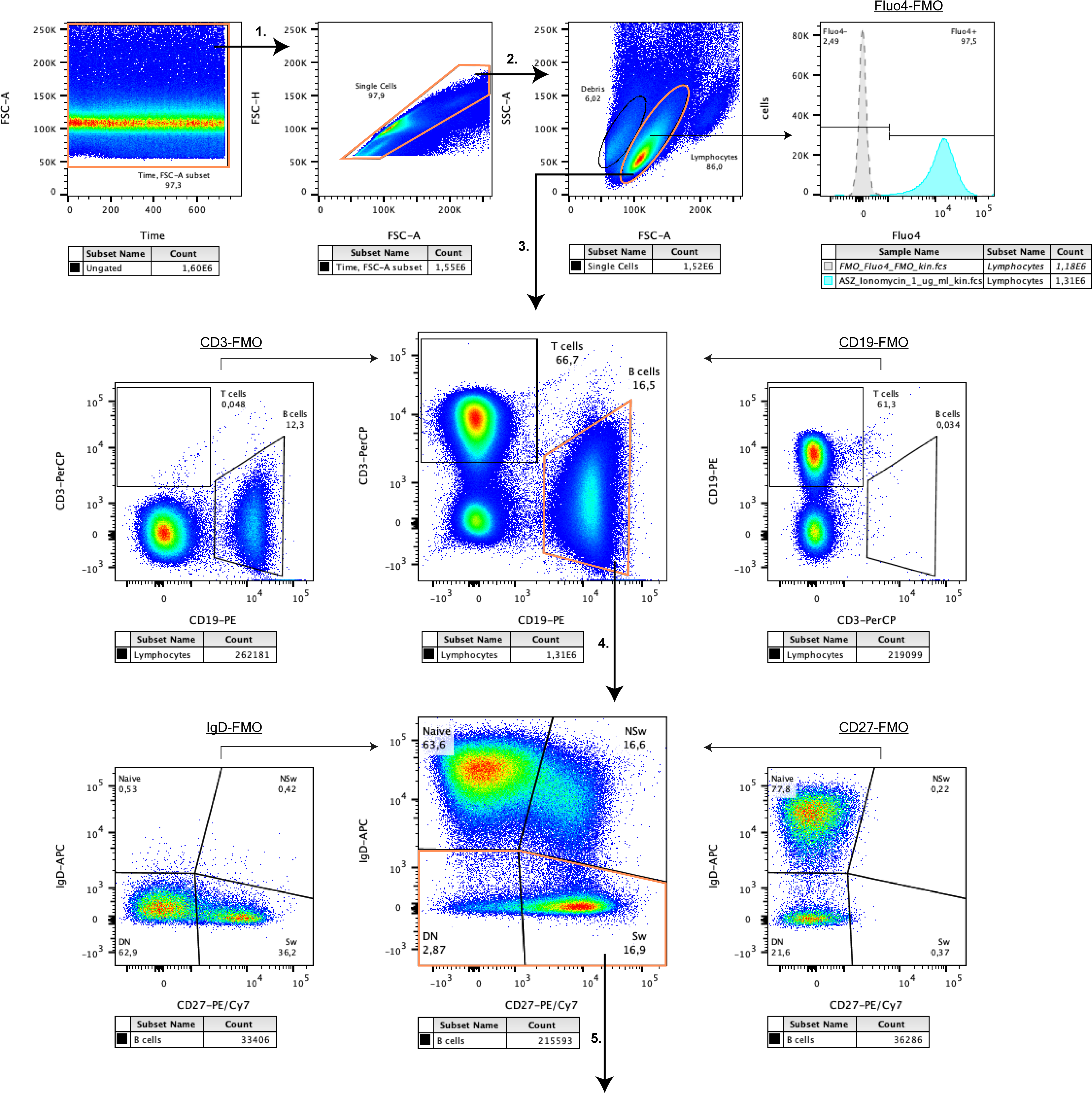

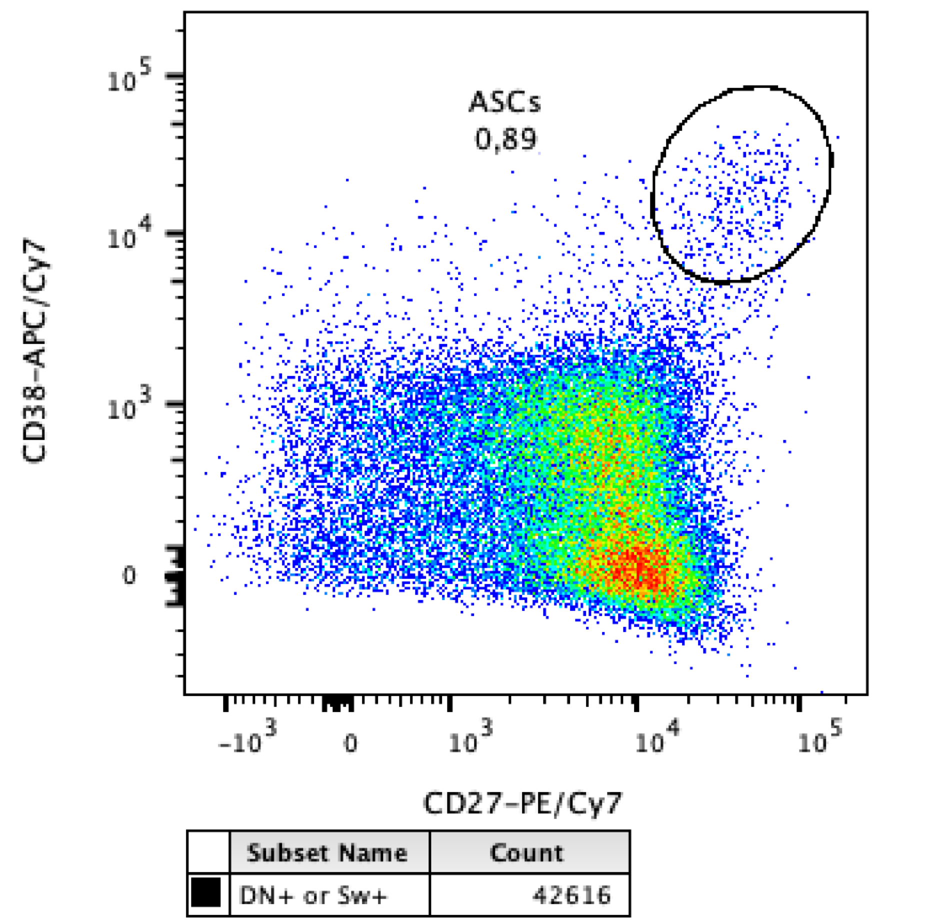

Supplementary Figure S1
Complete gating pathway with Fluorescence-minus-one (FMO) controls depicted as 2D pseudocolor dot-plots or single-parameter histogram. Orange-colored gates mark the parent population for the subsequent gating step: Time-Gate (**1.**) (Time / FSC-A)
→ Single cells (**2.**) (FSC-A / FSC-H)
→ Lymphocytes (**3.**) and Debris (FSC-A / SSC-A)
→ Fluo-4 positive (**4.**) and negative populations (Histogram of Fluo-4 fluorescent intensity) B cells (**5.**) and T cells (CD19-PE / CD3-PerCP)
→ Naive, Non-switched memory B cells (NSw), Switched memory B cells (Sw) and Double-negative memory B cells (DN) (CD28-PE/Cy7 /IgD-APC) → Boolean-gate (DN+ or Sw+) for IgD– B cells (**6.**)
→ Antibody-secreting cells (ASCs) (**7.**) (CD27-PE/Cy7 / CD38-APC/Cy7).

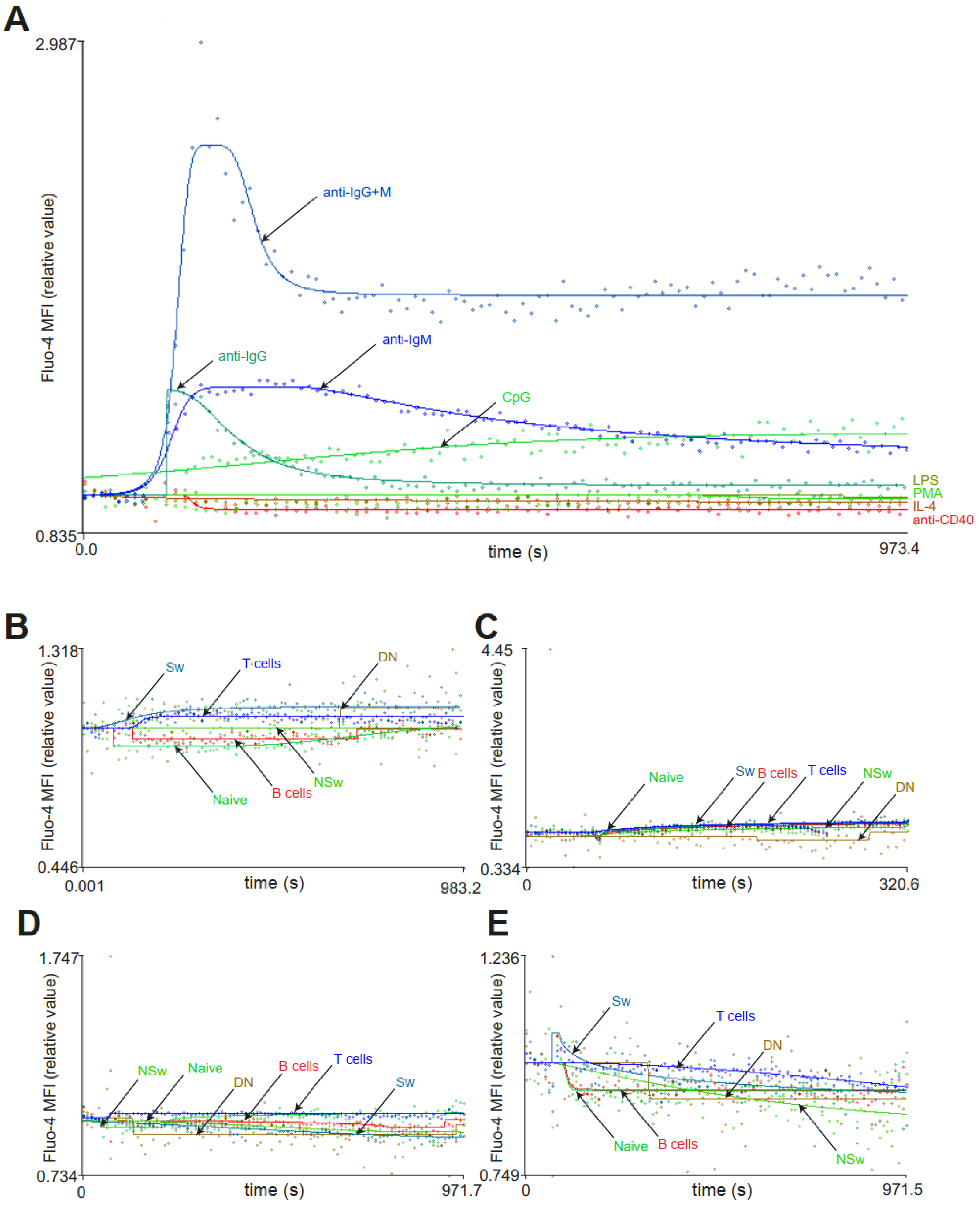

Supplementary Figure S2
Selection of investigated stimuli
Comparison of activating agents that trigger (anti-IgG+M, anti-IgG, anti-IgM, and CpG) to those that fail to trigger (LPS, PMA, IL-4, anti-CD40) a measurable Ca2+ response in B cells (**A**). Effect of LPS (**B**), PMA (**C**), IL-4 (**D**), and anti-CD40 (**E**) on the Ca2+ mobilization of different B cell subsets and T cells.
IgG/M = Immunoglobulin G/M; LPS = Lipopolysaccharide (O83); PMA = phorbol myristate acetate; IL-4 = Interleukin-4; Sw = switched memory B cells; DN = double negative memory B cells; NSw = non switched memory B cells.
